# A compact, *in vivo *screen of all 6-mers reveals drivers of tissue-specific expression and guides synthetic regulatory element design

**DOI:** 10.1186/gb-2013-14-7-r72

**Published:** 2013-07-18

**Authors:** Robin P Smith, Samantha J Riesenfeld, Alisha K Holloway, Qiang Li, Karl K Murphy, Natalie M Feliciano, Lorenzo Orecchia, Nir Oksenberg, Katherine S Pollard, Nadav Ahituv

**Affiliations:** 1 Department of Bioengineering and Therapeutic Sciences, University of California San Francisco, 1550 4th St, San Francisco, CA 94158, USA; 2 Institute for Human Genetics, University of California San Francisco, 1550 4th St, San Francisco, CA 94158, USA; 3 Gladstone Institutes, University of California San Francisco, 1650 Owens St, San Francisco, CA 94158, USA; 4 Division of Biostatistics, University of California San Francisco, 1650 Owens St, CA 94158, USA; 5 Department of Mathematics, Massachusetts Institute of Technology, 77 Massachusetts Ave, Cambridge, MA 02139, USA; 6 Current address: Institute for Pediatrics, Translational Research Center for Development and Disease, Children's Hospital of Fudan University, Shanghai, 201102, China

## Abstract

**Background:**

Large-scale annotation efforts have improved our ability to coarsely predict regulatory elements throughout vertebrate genomes. However, it is unclear how complex spatiotemporal patterns of gene expression driven by these elements emerge from the activity of short, transcription factor binding sequences.

**Results:**

We describe a comprehensive promoter extension assay in which the regulatory potential of all 6 base-pair (bp) sequences was tested in the context of a minimal promoter. To enable this large-scale screen, we developed algorithms that use a reverse-complement aware decomposition of the de Bruijn graph to design a library of DNA oligomers incorporating every 6-bp sequence exactly once. Our library multiplexes all 4,096 unique 6-mers into 184 double-stranded 15-bp oligomers, which is sufficiently compact for *in vivo *testing. We injected each multiplexed construct into zebrafish embryos and scored GFP expression in 15 tissues at two developmental time points. Twenty-seven constructs produced consistent expression patterns, with the majority doing so in only one tissue. Functional sequences are enriched near biologically relevant genes, match motifs for developmental transcription factors, and are required for enhancer activity. By concatenating tissue-specific functional sequences, we generated completely synthetic enhancers for the notochord, epidermis, spinal cord, forebrain and otic lateral line, and show that short regulatory sequences do not always function modularly.

**Conclusions:**

This work introduces a unique *in vivo *catalog of short, functional regulatory sequences and demonstrates several important principles of regulatory element organization. Furthermore, we provide resources for designing compact, reverse-complement aware *k*-mer libraries.

## Background

The activity of regulatory elements is governed by transcription factors (TFs), which bind to DNA sequences averaging 6 to 9 bp in size [1] and function in combination to achieve diverse gene expression patterns. Knowledge of the vertebrate TF repertoire and individual TF binding site (TFBS) preferences is still far from complete, due to binding-site degeneracy and the challenge of annotating functional TFBSs that may occur over a million base pairs from the transcription start site (TSS) of their target gene. Even less is known about the minimal functional elements that are sufficient to drive tissue-specific expression, and how elaborate patterns result from their interplay. Deciphering the vocabulary and grammar of the vertebrate regulatory code will enable high-resolution mapping of regulatory elements, accurate interpretation of nucleotide variation in regulatory sequences, and the design of regulatory elements to deliver genes to specific tissues for research and therapeutic purposes.

Many top-down approaches are used to predict vertebrate regulatory sequences. For a subset of vertebrate TFs, motifs derived from *in vitro *measurements of binding affinity [2,3] can be used to identify potential TFBSs in genomic sequences. Distinguishing which TFBSs are functional is challenging, even with the aid of evolutionary conservation [4,5] and clustering of TFBSs [6,7]. Techniques that combine biochemical assays with massively parallel sequencing, such as ChIP-seq, DNase-seq and formaldehyde-assisted isolation of regulatory elements (FAIRE)-seq, identify potential gene-regulatory elements by measuring TF binding and open chromatin in a specific cell type on a genome-wide scale [8-17]. However, these methods only coarsely locate functional sequences and are predictive in nature, requiring downstream validation. There is evidence that regulatory signals may be short (approximately 44 bp) [18], but canonical regulatory elements identified by these techniques tend to be quite long. For example, the average size of the 846 functional elements currently listed in the VISTA enhancer browser is 1,812 bp [19]. Traditional efforts to bash larger elements for core domains [18] are effective, but are time-consuming and costly.

To complement existing methods for identifying regulatory regions, it will be essential to reverse-engineer regulatory elements to reveal the fundamental units that drive complex expression patterns and the rules that govern their organization. One study that employed such an approach [20]*in vitro *tested 100-bp synthetic elements, each composed of tandem repeats of multiplexed 10-bp sequences, in six cell lines. Considerable differences in expression levels were observed between cell lines, consistent with the tissue-specificity of native vertebrate regulatory elements [8]. However, *in vivo *assays are essential to understanding how the vertebrate regulatory logic functions in a physiological environment involving multiple cell types, cellular interactions and temporal stages.

Here, we developed a bottom-up approach to characterize regulatory element vocabulary by systematically cataloging the effect of each 6-bp sequence (6-mer) on a TATA-box-containing 42-bp minimal promoter using zebrafish transgenics. To make this comprehensive screen feasible *in vivo*, we multiplexed the 6-mers into an ultra-compact library of 184 double-stranded constructs, each 15-bp in length. The activity of each construct was then scored in 15 tissues at 24 and 48 hours post-fertilization (hpf). We identified 27 multiplexed oligomers (15%) that produced distinct, consistent expression patterns. Four such constructs with distinct expression profiles were functionally dissected to identify even shorter 9-bp sequences that match binding motifs of known TF families, suggesting a mechanism by which they achieve tissue-specificity. Removal of these functional sequences from native zebrafish enhancers reduced their activity. Functional sequences were also used to design larger completely synthetic homotypic regulatory modules that drove strong expression to specific tissues. In contrast, heterotypic combinations of synthetic concatemers revealed a non-modular grammar.

## Results

### Design of an ultra-compact library of multiplexed 6-mers

**Figure 1:**
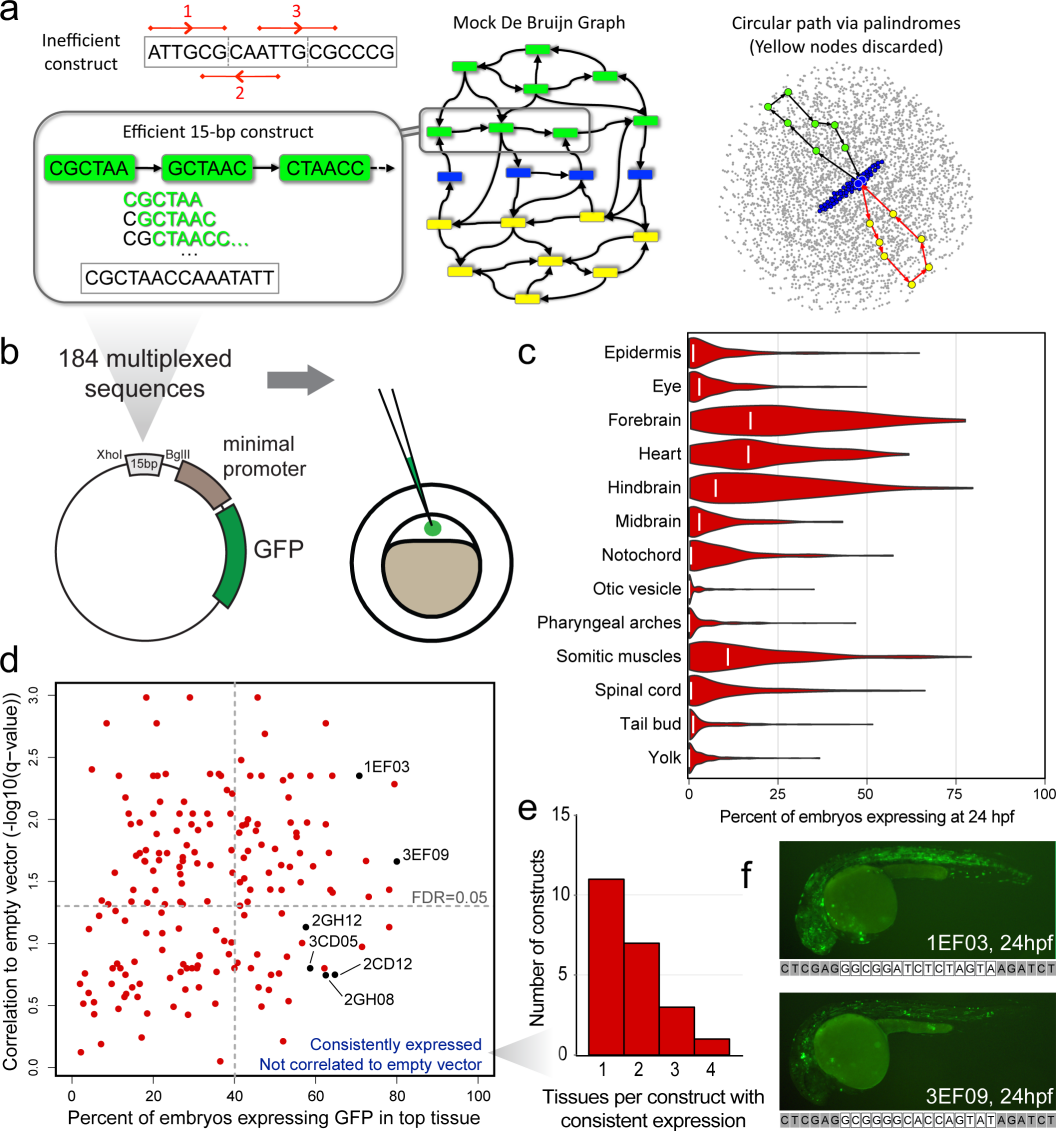
***In vivo *regulatory screen of all 6-mers**. **(a) **Left top: naively concatenating three 6-mers creates an oligomer with multiple representatives of ATTGCG (red bars). Center and left: cartoon of a de Bruijn graph. Nodes (colored boxes) represent 6-mers; edges (arrows) represent overlap. A standard de-Bruijn-sequence library is built from one path that traverses each of 4,096 nodes once. Constructed from multiple paths, our MRCC library instead uses one representative for each pair of reverse-complementary 6-mers (green and yellow; self-reverse-complementary palindromes in blue), making it nearly 50% smaller (Additional file 1). Right: 16 of 16,384 edges shown. Our algorithm removes reverse-complementary paths (black, red) between palindrome pairs and then decomposes the remaining graph into reverse-complementary cycles. It allowed us to design an ultra-compact library of DNA sequences with uniform 6-mer coverage. **(b) **Schematic depicting the sub-cloning of each 15-bp multiplexed oligomer into the E1b-Tol2 vector and subsequent injection into single-cell zebrafish embryos. **(c) **Violin plots depicting the distribution of the expression patterns of each tissue at 24 hpf. White lines indicate the fractional expression values for the empty vector construct. **(d) **Scatter plot depicting the method by which we selected consistently expressed multiplexed oligomers whose expression was not significantly correlated to minimal-promoter bias. The vertical dotted line denotes the 40% fractional expression threshold that was used, whereas the horizontal dotted line corresponds to a false discovery rate-adjusted *P*-value of 0.05. **(e) **Histogram depicting the tissue specificity of the 22 uncorrelated, consistently expressed constructs at 24 hpf. **(f) **Representative images at 24 hpf for embryos injected with 1EF03 (top) and 3EF09 (bottom), exhibiting broad expression that was correlated with that of the empty vector expression. The full sequence of each construct (with XhoI and BglII flanking sites) is listed below each figure. Both constructs have a 5' GC-rich region. GFP, green fluorescent protein.

The number *n_k _*of unique sequences of length *k *(*k-*mers) is 4*^k^*, and therefore, values in the TFBS-length range of 6 to 9 bp produce an infeasible number of sequences for individual testing. On the other hand, values of *k *less than 6 would not be expected to cover a substantial portion of the TFBS binding spectrum. A solution to this problem is to multiplex *k-*mers into longer oligomers. However, the length of the oligomers is inversely correlated with the information that can be learned about individual *k*-mers and proportional to the follow-up testing (for example, bashing) required for identifying the active *k-*mers. Additionally, inefficient multiplexing (Figure 1a), exemplified by concatenating *k*-mers or randomly generating oligomers, results in large libraries with biased *k*-mer coverage. By developing mathematical and computational techniques for efficiently multiplexing *k*-mers into oligomers (Additional file 1), we balanced these criteria at *k *= 6 (*n_k _*= 4,096) and an oligomer length of 15 bp.

Compact *k*-mer libraries have been used in several applications, such as protein-binding microarrays [3], universal DNA tag systems [21], and microfluidic affinity analysis devices [22]. This type of library is constructed from dividing up a de Bruijn sequence, which contains one copy of every *k*-mer. This sequence can be generated from a de Bruijn graph (Additional file 2), a mathematical object that has been applied in several biological contexts, particularly read assembly [23]. For *k *= 6 and a length of 15 bp, such a library would contain approximately 400 oligomers.

The theory underlying de Bruijn sequences was developed for general alphabets, not DNA. With the exception of self-reverse-complementary palindromes, each *k*-mer has a distinct reverse complement that is presumably equivalent to a TF binding double-stranded DNA. By restricting each reverse-complementary pair to a single representative on the forward strand, we reduced the library size by approximately 50%. Our design, which we call a minimal reverse-complementary-covering (MRCC) library of order *k *and size *m*, is a collection of *m *DNA sequences in which a single representative for each reverse-complementary pair of *k-*mers appears exactly once. To create an MRCC library, we designed an algorithm (Additional file 1) that computes a new type of palindrome-aware edge decomposition of the de Bruijn graph (Figure 1a) and enables us to account for reverse complementarity. In contrast to a de Bruijn sequence, which is obtained by computing a single cyclical path that traverses every edge in the graph exactly once (Additional file 2), an MRCC library is obtained by partitioning the edges into many paths. We note that for odd values of *k*, the algorithm is similar to a previous heuristic [24] that attempts to compute an analog to the de Bruijn sequence. However, this analog does not exist for even values of *k *larger than 2 (Additional file 1).

Using custom software that we have made available [25], we created an MRCC library of 208 DNA oligomers, each 15 bp, with uniform, single-copy coverage of all 6-mers. By exploiting 6-mers that overlap junctions between oligomers and flanking sequence, a modified version of the algorithm reduced the number to 184 (Additional file 3). This library is about 45% of the minimum size obtainable with previous algorithms that give uniform coverage.

### Multiplexed oligomer screen identifies functional units driving tissue-specific expression

Each of the 184 multiplexed 15-bp oligomers was synthesized and cloned upstream of the 42-bp adenovirus type 5 E1b [26] minimal promoter and green fluorescent protein (GFP) in a Tol2 retrotransposon-based [27] zebrafish reporter vector [28]. Each construct was injected into single-cell zebrafish embryos, and GFP expression was monitored in 15 tissues at 24 and 48 hpf (Figure 1b). These time points coincide with the establishment of the basic vertebrate bauplan and the development of key structures. In addition, embryos were injected with an empty vector control (identical flanking sequence but no insert) to assess the contribution of the minimal promoter. A substantial portion of the MRCC library (70/184 constructs, approximately 38%, at 24 hpf) drove consistent (in >40% of embryos) expression patterns, though our design was unguided by *a priori *knowledge and only a small amount of information could be encoded in each oligomer. A potential explanation for this is that our constructs, like regulatory regions marked selectively by histone 3 lysine 4 mono-methylation (H3K4me1), have a higher GC content than the zebrafish genome (average 43.1% for H3K4me1 and 50% for our multiplexed oligomers, versus 36.4% genome-wide). An alternative explanation is that while the oligomers are very short, the design of our MRCC library encodes a maximum amount of sequence diversity. It is thus likely that the MRCC library contains more active sequences than random genomic sequence of the same size. Our results are consistent with a much smaller percentage of the genome being active in regulation.

We observed a diverse range of expression at both time points (Figure 1c; Additional files 2 and 3). However, there was a clear bias towards amplifying the background bias of the minimal promoter (Figure 1c; Additional file 2). By removing expression patterns that were significantly correlated with background expression (Spearman rank correlation coefficient, false discovery rate (FDR) q < 0.05), we identified 22 multiplexed constructs that drove GFP expression consistently at 24 hpf to at least one tissue (Figure 1d; Additional file 3). An additional five constructs produced uncorrelated and consistent expression solely at the 48 hpf time point (Additional file 2).

Tissue specificity is a common feature of regulatory elements [29]. We thus examined the tissue distribution of constructs expressed consistently in one or more tissue. Eleven out of the 22 constructs (50%) that produced consistent and uncorrelated expression at 24 hpf did so in a single tissue (Figure 1e). In contrast, several correlated constructs exhibited very broad expression patterns. For example, 1EF03 and 3EF09 produced the strongest observed signal in ten tissues across the two time points, likely due to a GC-rich sequence embedded at the 5' end of each oligomer (Figure 1f).

**Figure 2:**
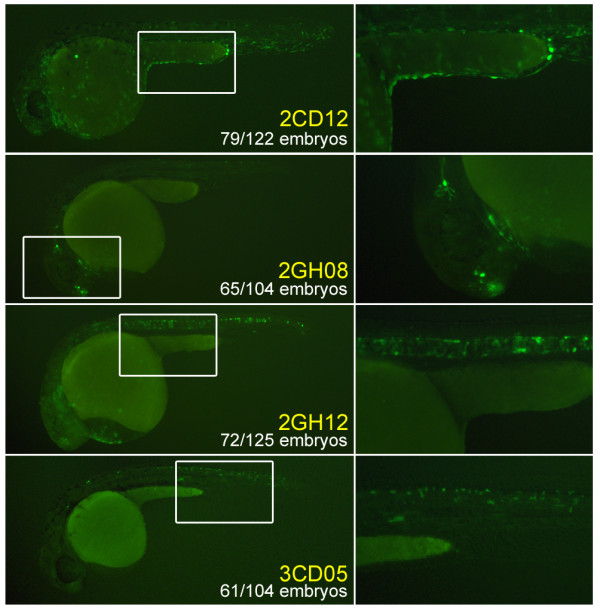
**The four uncorrelated constructs chosen for follow-up experiments due to their consistent, specific expression patterns: 2CD12/epidermis; 2GH08/brain (showing both forebrain and otic lateral line expression); 2GH12/notochord; 3CD05/spinal cord**. White rectangles indicate regions that are magnified in the right side panels.

We chose a subset of the consistently expressed, tissue-specific, uncorrelated constructs to pinpoint functional sequences within the constructs and the zebrafish genome: 2CD12/epidermis, 2GH08/brain, 2GH12/notochord, and 3CD05/spinal cord (Figure 2). 2GH08/brain was unique in this set in that it expressed consistently in more than one tissue: bilaterally in rostral forebrain neurons at both time points and in ganglia of the otic lateral line at 24 hpf. For comparison, representative negative embryos injected with the empty vector control are depicted in Additional file 2. We confirmed that the expression pattern of these four constructs was due to genomic integration by producing F1 transgenics. In each case, F1 GFP expression resembled that of F0 embryos (Additional file 2). To address the dependence of each sequence on the E1b minimal promoter, we swapped it with another TATA-box containing 31-bp element commonly used in luciferase reporter assays (see Materials and methods). Three of the four constructs had essentially identical patterns, suggesting that the observed tissue-specific activity is guided by the 15-bp sequence (Additional file 2). 2GH12/notochord was the outlier, suggesting that a sequence within the E1b minimal promoter could be needed for its tissue specificity.

### Multiplexed oligomers contain shorter, functional sequences

A previously reported machine-learning algorithm assigned weights to 6-mers based on their association with putative forebrain enhancers [6], as defined by the binding of the E1A binding protein p300 (EP300) in mouse forebrain at embryonic day 11.5. We used these forebrain enhancer weights to score each of the 184 multiplexed constructs based on the combined weight of all 6-mers it contains. Higher scores indicate constructs with sequence patterns that are positively associated with EP300 binding in embryonic mouse forebrain. Using a logistic regression model, we compared the scores of constructs that had 24 hpf forebrain expression in the top 10% of our study with the scores of the remainder of the constructs. This analysis identified a significant association between our top forebrain constructs and 6-mers identified by Lee *et al*. (odds ratio = 1.17, *P *= 0.008; Wald test of logistic regression coefficient), despite differences in methods, organism, and developmental stage between the two studies. We found an even stronger association using the top 5% of the forebrain constructs (odds ratio = 1.23, *P *= 0.008; Wald test).

**Figure 3:**
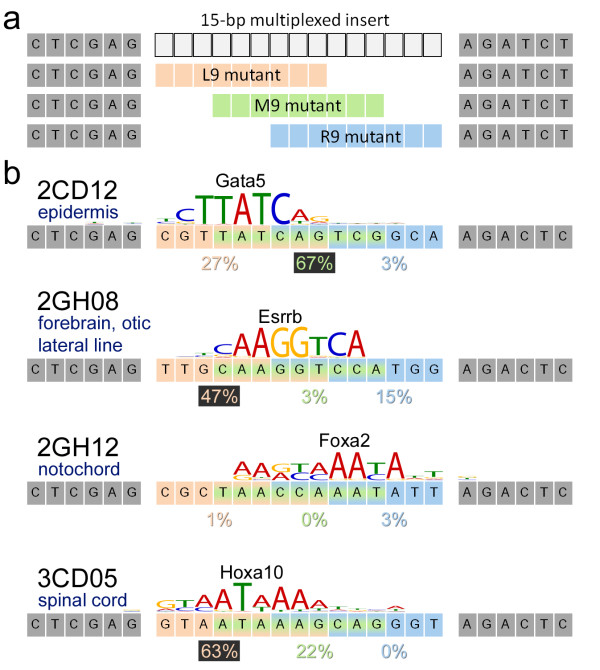
**Functional dissection of multiplexed sequences reveals key motifs**. **(a) **We tested three deletion mutants for each of the four tissue-specific multiplexed sequences, using the first (L9, peach), middle (M9, green) and last (R9, blue) 9 bp of each sequence cloned between the same flanking sequences. **(b) **With one exception (2GH12/notochord), a 9-bp sequence was sufficient to drive the expression pattern of the original multiplexed construct. Percentages of fish expressing in the appropriate tissue at 24 hpf are shown, with the value for the functional 9-mer highlighted in black. The top-matching position weight-matrix identified by Tomtom [30] is listed for each construct.

For the 18 multiplexed oligomers with forebrain expression in the top 10% of our study, an average of only 8 of 14 distinct 6-mers in each construct have positive weights. This suggests that multiple 6-mers in each construct are dispensable for forebrain expression. To functionally explore this hypothesis in multiple tissues, we performed a functional dissection experiment on the four selected tissue-specific constructs. For each, we removed 6 bp to leave the left (L9), middle (M9), and right (R9) 9 bp of each 15-bp insert (Figure 3a). In all but one case (2GH12/notochord), a single 9-bp sequence was sufficient to largely reproduce the expression pattern of the original multiplexed construct (Figure 3b; Additional file 4).

We used Tomtom [30] to identify JASPAR [31] and UniPROBE [32] position weight matrices (PWMs) that were similar to the functional 9-bp (or 15-bp, in the case of 2GH12) sequences identified through the functional dissection experiments. Although TF binding specificities are thought to be conserved, the PWMs in these databases are not derived from zebrafish TF binding specificities, and thus are not ideal. However, in each case, the top hit (Figure 3b) suggested TFs or TF families that plausibly interact with these sequences. The M9 region of 2CD12/epidermis contains a GATA motif on the reverse strand, matching the UniPROBE PWM for Gata5 (*P *= 0.001, Tomtom empirical *P*-value [30]) but likely corresponding to Gata3 in zebrafish, which binds to a very similar motif *in vitro *(Additional file 2) and is a regulator of epidermal cell lineage [33]. Likewise, the L9 region of 3CD05/spinal cord matches the UniPROBE PWM for Hoxa10 (*P *= 0.0005), although this motif likely is recognized by a more medially expressed hox in zebrafish, such as Hoxa9a. Mammalian Hoxa10 and Hoxa9 binding motifs are extremely similar, and *hoxa9a *expression precisely mirrors the activity of 3CD05/spinal cord (Additional file 2). The L9 region of 2GH08/brain strongly matches the JASPAR PWM for Esrrb (*P *= 0.001), the zebrafish homolog of which is expressed in forebrain and hindbrain nuclei, recapitulating the expression pattern driven by the 15-bp multiplexed oligomer (Additional file 2). 2GH12/notochord strongly matches the UniPROBE PWM for Foxa2 (*P *= 0.0009), which is expressed in the zebrafish notochord at the Prim-5 stage (approximately 24 hpf) [34]. The longer length of this motif provides a clue as to why 2GH12 could not be functionally dissected. In summary, the functional dissection experiments suggest the expression patterns of the four tissue-specific multiplexed oligomers are largely due to the action of shorter, 6- to 9-bp sequences.

### Design of synthetic regulatory elements from smaller functional units

**Figure 4:**
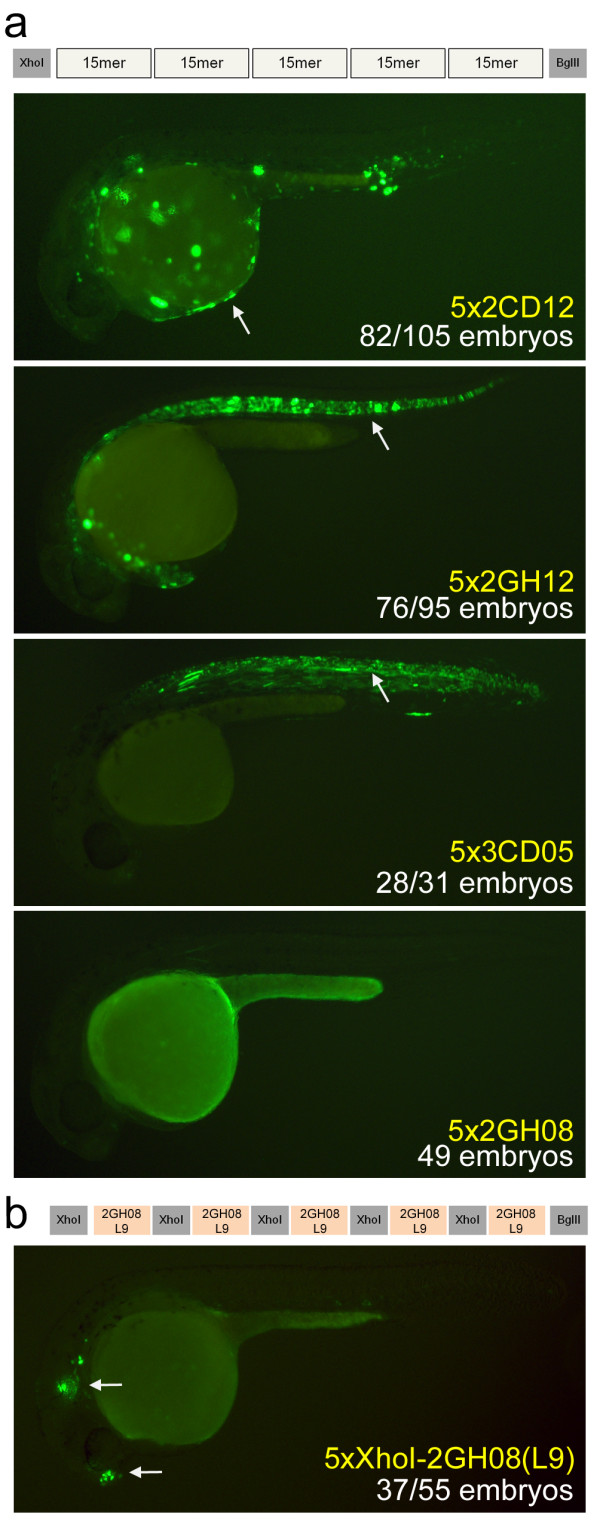
**Design of synthetic enhancers from short multiplexed sequences**. **(a) **We generated concatemers containing five copies of each of the six multiplexed sequences. Three of these (5x2CD12/epidermis, 5x2GH12/notochord, 5x3CD05/spinal cord) drove more intense expression in the same tissue as the original 15-bp sequence. The fourth construct, 5x2GH08/brain, did not produce any detectable expression, suggesting that the XhoI-flanking sequence may be important. White arrows mark the epidermis, notochord, and spinal cord, respectively. **(b) **A second version of the 2GH08/brain concatemer contained five tandem repeats of the XhoI flanking region with the 2GH08(L9) functional sequence that was identified by the 9-bp dissection experiments. This arrangement produced a strong regulatory element that drives consistent expression to the forebrain and otic lateral line at 24 and 48 hpf. White arrows mark the forebrain and otic lateral line patterns. All pictures are of 24 hpf embryos.

Regulatory sequences often contain clusters of homotypic TFBSs, with an average of five per cluster [35]. To determine whether this principle could aid in the design of synthetic regulatory elements, we generated concatemers of each of the four tissue-specific sequences, with five copies of each 15-bp insert, and tested them in zebrafish. 5x2CD12/epidermis, 5x2GH12/notochord and 5x3CD05/spinal cord produced more intense and specific expression patterns than their single-copy counterparts (Figure 4a; Additional file 2). In contrast, concatenation of 2GH08/brain abolished specific expression in the forebrain and otic lateral line entirely (Figure 4a). Combined with the functional dissection result, these data suggested that the XhoI-flanking sequence is necessary for proper function of the 2GH08 sequence. In a second round of concatemer design, we concatenated repeats of the functional 9-bp sequence from the functional dissection experiments (L9) along with the 6-bp XhoI flanking region. The construct XhoI-2GH08(L9) produced consistent expression (67% of embryos expressing) in rostral forebrain and otic lateral line in a more intense and specific pattern than 2GH08/brain (Figure 4b; Additional file 2). These results demonstrate that the strength of a regulatory element can be tuned by simple repeats of short, key regulatory sequences *in vivo*.

**Figure 5:**
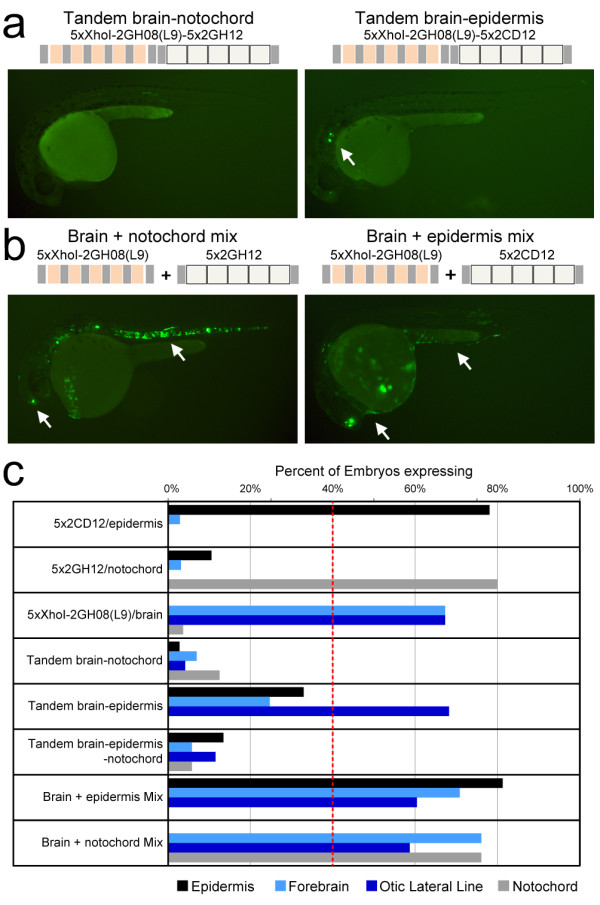
**Combination of synthetic regulatory elements underscores the importance of context**. **(a) **Left: a tandem combination of the 5xXhoI-2GH08(L9)/brain and 5x2GH12/notochord concatemers failed to produce any enhancer activity above background. Right: a tandem combination of the 5xXhoI-2GH08(L9)/brain and 5x2CD12/epidermis concatemers resulted in consistent otic lateral line expression. Expression in the forebrain and epidermis was detected inconsistently in a handful of embryos (such as the one shown), but not as intensely as the original concatemer. **(b) **Injection of a mixture of the 5xXhoI-2GH08(L9)/brain and 5x2GH12/notochord (left) or 5xXhoI-2GH08(L9)/brain and 5x2CD12/epidermis constructs (right) resulted in the expected combinatorial pattern (white arrows). **(c) **Percentage of embryos expressing in the epidermis, forebrain and notochord for the 5x concatemers individually, as combinations or as a mixture of two constructs. Tandem arrangement of concatemers in the same regulatory element largely failed to reproduce the expression patterns driven by concatemers individually or in a mixture, with the exception of the brain-epidermis combination, which preserved otic lateral line expression.

Large, distal-acting enhancers (average length 1,529 bp) have been shown to autonomously drive diverse expression [36]. However, the internal circuitry of individual enhancers is much more complex, requiring the precise spacing and ordering of regulatory sequences [37-40]. To assess the syntactic rules that apply at the scale of our synthetic regulatory elements, we assayed tandem combinations of two or three concatemers. A tandem combination of brain (5xXhoI-2GH08(L9)) and skin (5x2CD12) concatemers abolished expression in skin and forebrain but not the otic lateral line. A tandem combination of brain and notochord (5x2GH12) concatemers, as well as a tandem brain, skin and notochord triplet abolished regulatory element activity in those tissues (Figure 5a,c). In contrast, injecting pairs of concatemers as separate constructs yielded consistent, combinatorial expression of the two patterns (Figure 5b,c). In each tandem combination, the XhoI-2GH08(L9)/brain concatemer was the most distant from the minimal promoter, while the intervening sequence and the distance to the minimal promoter varied. The fact that two intervening sequences abolished the activity of the brain concatemer (brain+notochord and brain+skin+notochord), but another (brain+skin) only limited its spatial extent, suggests the local context of a regulatory element is essential to its function. These data are consistent with the idea that large, autonomous enhancers contain insulator elements and/or signals mediating long-range interactions [37] that are absent in our synthetic regulatory elements.

### Short, tissue-specific oligomers are essential components of native regulatory elements

**Figure 6:**
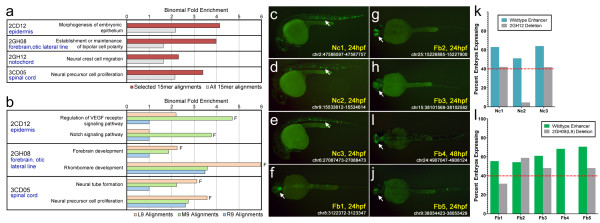
**Short sequences contribute to endogenous developmental zebrafish enhancers**. **(a,b) **Alignments of 15-bp multiplexed sequences and 9-bp sequences identified by functional dissection were intersected with predicted enhancers (defined by H3K4me1-positive, H3K4me3-negative ChIP-seq regions [12]). We then used GREAT [41] to find enriched ontology terms for nearby genes. In each case, a term consistent with the expression pattern was identified, suggesting that the sequences identified by our screen are performing specific roles in developing vertebrates. Plotted are binomial fold enrichment values for the specific 15-bp or 9-bp alignments. Functional 9-bp sequences are depicted by the letter F. To control for common developmental gene ontology terms, we include the binomial fold enrichment values for the H3K4me1+/me3- alignments of all 184 constructs (a) or the non-functional 9-bp sequences identified by functional dissection (b). We tested enhancer activity of 20 1,000-bp regions from the zebrafish genome that overlap with H3K4me1/me3- signal and contain an alignment for 2GH12/notochord or the 2GH08(L9)/brain sequence. **(c-e) **Three of ten 2GH12/notochord-containing regions (Nc1-3) showed strong notochord (Nc) enhancer expression at 24 hpf. **(f-j) **Five of ten 2GH08(L9)/brain-containing sequences (Fb1-5) were strong forebrain (Fb) enhancers at 24 or 48 hpf. The chromosomal coordinates of each enhancer are indicated (Zv9 zebrafish genome assembly) as well as the time point with the strongest expression. **(k,l) **Deletion of the 2GH12/notochord or 2GH08(L9)/brain sequence from the 1,000-bp enhancers leads to a reduction and, in several cases, abolition of the observed enhancer signal. Bars represent the percentages of fish exhibiting expression in the notochord (k) and forebrain (l) at the indicated time point provided in the pictures above. The red dotted line represents the 40% consistency cut-off used for this study.

To explore the native function of the multiplexed sequences in the zebrafish genome, we identified construct alignments in H3K4me1-positive, H3K4me3-negative predicted-enhancer regions [12] and then employed GREAT [41] to identify significantly enriched gene ontology terms for each construct. For each of the four 15-bp constructs, we identified one or more significantly enriched biological processes that were relevant to the tissue expression pattern (binomial test, FDR q < 0.05; Figure 6a; Additional file 5). We further aligned 9-bp sequences identified in the functional dissection experiments and performed the same analysis in GREAT, identifying several additional enriched terms that matched the corresponding expression pattern of the functional 9-bp sequence (Figure 6b; Additional file 6).

To determine if these sequences contribute to zebrafish regulatory modules, we picked ten 2GH12/notochord and ten 2GH08(L9)/brain alignments that overlap 24-hpf whole-embryo H3K4me1+/me3- ChIP-seq peaks. We cloned approximately 1,000-bp fragments, including the alignment and overlapping H3K4me1 signal, and tested them for enhancer activity in zebrafish. Three 2GH12- and five 2GH08(L9)-containing fragments drove consistent (>40% of fish expressing) expression to notochord and forebrain, respectively (Figure 6c-j; Additional file 7). A random selection of ten H3K4me1+/me3- regions identified two strong forebrain enhancers and two weak enhancers, driving expression consistently yet nonspecifically to the notochord and forebrain, respectively (Additional files 2 and 7). Together, these results indicate that these multiplexed sequences could be used in conjunction with other genome-wide datasets to improve the identification of functional, tissue-specific enhancers.

To determine the relative strength with which our synthetic elements can drive expression, we performed quantitative PCR on pools of 25 GFP-positive embryos injected with the original 15-bp insert, the 5x concatemer, or a strong native enhancer (Nc2/Fb2) corresponding to 2GH08/brain or 2GH12/notochord (Additional file 2). In each case, the native enhancer produced higher GFP expression than both the 15-bp insert (3.0- and 10.4-fold for brain and notochord, respectively) and the 5x concatemer (2.5- to 5.0-fold, respectively), suggesting that synthetic 15-mers and 5x concatemers are not as strong as native enhancers.

We next performed deletion mutagenesis on each positive 1,000-bp enhancer, removing the 2GH12/notochord- and 2GH08(L9)/brain-aligned regions to determine if they are required to drive the specific expression patterns observed. The deletions reduced the specific expression of the enhancer in seven out of eight cases and abolished it for one of three positive 2GH12- and two of five positive 2GH08(L9)-containing enhancers (Figure 6k,l; Additional file 7). These results demonstrate that the multiplexed regulatory sequences identified by our screen are necessary for the proper spatiotemporal expression of specific enhancers in the developing zebrafish.

## Discussion

In this study, we made several observations on the role of TFBS-sized sequences in gene regulation *in vivo*. First, we identified a considerable number of very short DNA sequences that are drivers of consistent, tissue-specific expression. A previous report carried out in various cell lines [20] suggested, as a rationale for screening concatemeric sequences, that such short sequences could not drive observable levels of transcription. Our results suggest that this is not the case, and that there exists an intermediate stage between neutral sequence and fully-fledged regulatory elements. We also showed that expression levels can be amplified *in vivo *through homotypic concatenation, a principle that has previously been demonstrated primarily in *in vitro *transient transfections [38,42,43]. Together, these findings suggest a potential mechanism through which short sequences could gain a toehold as minimal regulatory elements and evolve into more complex clusters.

A limitation of this approach is that each multiplexed construct contains multiple *k*-mers, requiring further bashing to determine functional sequences. However, such functional dissection is negligible compared to what would be required for a typical enhancer of more than 1,000 bp. Furthermore, multiplexing 6-mers into 15-bp oligomers identified sequences such as 2GH12, which was the only construct to drive notochord-specific expression and could not be broken down into smaller, functional units. This result, in particular, points to the potential benefits of testing higher order *k*-mers, ideally without multiplexing.

We additionally demonstrate that short *k*-mers (9 to 15 bp) are important for the proper spatiotemporal expression of a subset of distal-acting regulatory elements. Thus, even though our multiplexed sequences were generated synthetically, they harbor endogenous signals that confer tissue-specificity. Using a straightforward TFBS analysis tool and publicly available data, we identified potential interacting proteins for all four tissue-specific signals. However, these matches were not perfect, highlighting the need for more *in vivo *testing of short sequences.

Another contribution of this work is a mathematical and computational framework for the design of ultra-compact DNA libraries with single-copy representation of pairs of reverse-complementary *k-*mers. We show here that the reverse complementarity of DNA can be exploited to construct an MRCC library, which is about half the size of the analogous de Bruijn sequence library and yet still maintains uniform, minimal coverage of *k*-mers. Traditional de Bruijn sequences have proven to be extremely useful for biological applications, including protein-binding microarrays [3], universal DNA tag systems [21] and microfluidic affinity analysis devices [22]. Our formal analysis of the structure of the de Bruijn graph induced by the reverse complementarity of DNA should aid in the advancement of these and other specialized techniques that use *k*-mer libraries [20,44]. In addition, the use of programmable microarrays to generate barcoded libraries of sequences for functional testing would provide an enticing platform for applying MRCC libraries. These 'massively parallel reporter assays' (MPRAs) have so far only been used to test variants of an individual promoter or enhancer [45-47], but could be used to screen *k*-mer libraries on a larger scale.

Extending such methodologies to higher *k *may aid in the discovery of drivers for tissues such as the eye, otic vesicle, and fins, which were poorly represented in the 6-mer screen. For these tissues, it is entirely possible that 6-mers are simply not sufficient to drive expression. Although our screen covered 100% of 6-mers, it only captures 32% of all 7-mers, 9% of all 8-mers, and a mere 2% of the 262,144 unique 9-mers. Such methods could be combined with transgenic lines with secondary markers for specific tissues of interest to improve annotations.

Finally, as evidenced by a homotypic concatemer whose expression is affected by downstream sequences, we show that local context affects enhancer function. Our findings are consistent with previous reports that show that the function of regulatory elements is sensitive to changes of spacing and order [37-40], but could also imply that our synthetic enhancers are missing key, yet undiscovered, signals typical of endogenous enhancers. While deciphering the rules of syntax by testing individual combinations is technically challenging, this process could also be accelerated by testing rearrangements of a known enhancer in parallel, using MPRAs. One such example is the recent use of MPRA technology to characterize the regulatory logic of promoters in yeast [43]. Such large-scale functional dissections will aid in the design of synthetic regulatory elements that can drive molecules to a specified level of expression in any combination of tissues and time points.

## Conclusions

Our unique catalog of short, functional sequences is a good starting point in the search for the basic functional units contributing to regulatory element activity. These sequences could be used to improve genome annotation and to interpret the effects of non-coding genetic variants. Another application is in the design of synthetic regulatory elements that can drive expression of molecules to specific tissues and time points. Using only a small subset of the 'words' in our 6-mer regulatory catalog, we demonstrate a simple type of 'sentence' that obeys the syntactical rule of amplification through repetition. Attempts to construct more elaborate sentences revealed a non-modular grammar at the scale of TFBS-sized elements, highlighting the need for functional assays with higher throughput.

## Materials and methods

### Ultra-compact 6-mer library design

Although we chose *k *= 6, our methods are applicable in principle to larger values of *k *and libraries of varying size. We restricted the design to include just one *k*-mer out of each reverse-complementary pair of *k*-mers ('*k*RC-pair'). For even values of *k*, a *k*RC-pair may consist of a single self-reverse-complementary palindrome, rather than two sequences. For an RC-pair *X *and collection *S *of *m *DNA sequences, χ(*X*,*S*) denotes the total number of times that the *k-*mers in *X *appear as substrings in all sequences of *S*. For example, if *X *= {ATTTTA, TAAAAT} and *S *= {GATTTTATTTTAGGG, TAAAATGGATTTTAA}, then χ(*X*,*S*) = 4. We introduced the concept of a MRCC library of order *k *and size *m*, which, formally, is a collection *S *of *m *DNA sequences such that for every *k*RC-pair *X*, χ(*X*,*S*) = 1.

The *k-*dimensional de Bruijn graph (Figure 1a; Additional file 2) has *n_k _*vertices, each labeled by a unique *k*-mer, and *n_k_*_+1_directed edges, each labeled by a unique (*k*+1)-mer. The edge labeled by *x *is directed from the vertex labeled by the first *k *bases of *x *to the vertex labeled by the last *k *bases of *x*. Every path, i.e., sequence of edges, corresponds to a DNA sequence: the label of the first edge gives the first *k*+1 bases of the sequence, and for *i *> 2, the final base in the label of the *i-*th edge gives the (*k*+*i*)-th base in the sequence. The reverse complement of a path *t *is also a path: it consists of the reverse complements of the edges in *t*, traversed in reverse order. The (*k*-1)-dimensional de Bruijn graph is used to efficiently generate a de Bruijn sequence of order *k *via the computation of an Eulerian cycle, that is, a single cyclical path that traverses every edge in the graph exactly once (Additional file 2). There is an equivalent path that traverses every node exactly once in the more visually intuitive *k*-dimensional de Bruijn graph, described in Figure 1a.

To create an MRCC library, we designed an algorithm (Additional file 1) that partitions the edges of the de Bruijn graph such that there are at most two palindromes per path (Additional file 2). Our algorithm has two main stages. Stage 1, which is only executed for even values of *k *(*k *> 2), iterates over pairs of palindromes, removing from the graph two reverse-complementary paths between each pair. The paths correspond to reverse-complementary sequences, one of which is added to the library. Stage 2 partitions the remaining edges into two reverse-complementary cycles. The sequence that corresponds to one of those cycles is then divided into the remaining number needed of consecutive oligomers such that the final *k*-1 bases of each sequence overlap the first *k*-1 bases of the next (Additional file 2). The overlap is required to maintain *k*RC-pair coverage. These oligomers complete the library.

The algorithm is defined for any integers *k *and *m *such that *k >*0 and ½*p_k _<m <*(½*n_k _*- ¼*kp_k_*) (for *k *= 2, the range is 0 *<m <*½*n_k_*). For *k *= 6, *m *must be between 32 and 1,952, which meets the constraints of our construct design, which required *m *to be a feasible number for *in vivo *testing.

Stage 2 of our algorithm, which is an adaptation of the classic Eulerian-cycle algorithm by Hierholzer [48], is similar to a heuristic algorithm sketched by Mintseris and Eisen [24] that attempts to partition all edges of the (*k*-1)-dimensional de Bruijn graph into just two reverse-complementary cycles. For even values of *k *larger than 2, it is not possible to partition the edges this way; every MRCC library has size *m *> 1 (Additional files 1 and 2). For odd values of *k *and *k *= 2, this edge partitioning does exist (Additional file 2); one of the two cycles is equivalent to an MRCC sequence.

If the initial bases of an oligomer insert match the final bases of the left flanking sequence, then those bases can be eliminated from the oligomer to reduce its length without sacrificing its *k*RC-pair coverage. The same is true for the final bases of the oligomer and the right flanking sequence. We created a modified version of the algorithm that uses heuristics during cycle-finding and sequence-splitting to produce oligomers with these characteristics. This technique further reduced library size and the number of *k*-mers repeated across the junctions between oligomers and flanking sequences.

### Software implementation

We implemented the original algorithm and used it to produce an initial construct design for *k *= 6 that contains *m *= 208 oligomers, each of length 15 bp. The number *N_k _*of *k*RC-pairs is *N_k _*= *p_k _*+ (½)(*n_k _*- *p_k_*) = (½)(*n_k _*+ *p_k_*), where *n_k _*= 4*^k ^*is the number of *k*-mers and *p_k _*= 4*^k/2 ^*is the number of palindromes. For *k *= 6, *N_k _*= 2,080, *n_k _*= 4,096, and *p_k _*= 64, which implies that the initial design is optimally compact, excluding flanking sequences. With our implementation of the flanking-sequence-based, modified version of the algorithm, we reduced the number of oligomers by more than 10%, from 208 to 184, still each 15 bp.

Due to Perl's facility in handling sequences, all implementations are in Perl. They can be adapted for better time and space efficiency, if needed for larger values of *k*, using a compiled programming language, such as C, and specific data structures (Additional file 1). All code is available from [25].

### Plasmids and constructs

For the primary screen and deletion series, each oligomer (Additional file 3) and its reverse complement were synthesized (Bioneer Inc., Daejeon, Republic of Korea) with 5' and 3' overhangs, annealed and then cloned between XhoI and BglII restriction sites of the zebrafish enhancer vector E1b-Tol2 [28]. For the endogenous enhancer screen, 1,000-bp regions were amplified from zebrafish genomic DNA using CACC flanked primers, and then cloned into pENTR using the TOPO Cloning kit (Life Technologies, Carlsbad, CA, USA). Cloned enhancers were subsequently transferred to the E1b-Tol2 vector using the LR Clonase II cloning enzyme (Life Technologies). For the concatemeric constructs, 150- to 225-bp regions were synthesized and cloned into E1b-Tol2 (Biomatik USA, LLC, Wilmington, DE, USA).

### Zebrafish injections

Constructs were injected with Tol2 by standard techniques [27,49]. An average of 60 embryos were injected for the primary screen of 184 oligomers, with the secondary requirement that at least 25 embryos survived to 48 hpf. The expression patterns of the four tissue-specific constructs chosen for follow-up experiments were monitored in at least 100 embryos at each time point. For the alternative minimal promoter screen, we replaced the E1b minimal promoter with the 31-bp minimal promoter region from the pGL4.23 luciferase reporter vector (Promega, Madison, WI, USA).

GFP expression was recorded in 13 tissues at 24 hpf and 15 tissues at 48 hpf according to established ZFIN nomenclature [50]. At least two images (one lateral, one dorsal or ventral) were acquired for each construct, with more images taken for constructs showing consistent expression patterns. A complete collection of screen data and a collection of image data for consistently expressing constructs are available from our website [51].

### Correlation analysis

For each multiplexed construct, fractional expression values were determined by dividing the number of embryos expressing in a given tissue by the total number surviving at each time point. The same values were obtained for an "empty vector" control construct lacking an oligomer insert. Correlation between these two profiles was then determined for each construct and time point using the Spearman's rank correlation coefficient. Constructs with an FDR-adjusted *P*-value <0.05 were considered correlated to the empty vector.

### Support-vector machine forebrain enhancer weights

Using the 6-mer weights assigned by a previously reported support vector machine (SVM) that predicts EP300-binding in forebrain [6], we computed a score for each construct by summing the weights of the 6-mers in the construct (15-bp oligomer insert with 2-bp flanking sequence on each end). We binarized our expression data, assigning 1 to a construct if its forebrain expression value was in the top 10% (or 5%) of all constructs at 24 hpf, and 0 otherwise. Those data were used to fit a logistic regression model using R [52], with construct score as the predictor variable. We tested for statistical significance of the association between the score and the probability of forebrain expression in the top 10% (5%) using a Wald test.

### Transcription factor binding site analysis

Nine or 15-bp (in the case of 2GH12) functional sequences were compared to known vertebrate JASPAR [31] and UniPROBE [32] PWMs using Sandelin-Wasserman similarity [53] in Tomtom [30]. The most statistically significant PWM match to each construct was identified.

### Alignments to the zebrafish genome

Fifteen-base-pair inserts were aligned to the zebrafish genome (build Zv9) using bowtie v0.12.730 [54], allowing three mismatches and masking repeats (UCSC Genome Browser [55] RepeatMasker track). For the shorter, functional sequences identified by the functional dissection experiments, we aligned 11-mers (9 bp + 2 bp of flanking sequence), with one mismatch allowed.

### Enrichment analyses

GC content was computed from the Zv9 assembly, with repeats masked. Enrichment analyses were performed using Fisher's exact test. *P*-values were Bonferroni adjusted.

We predicted enhancer regions using previously reported histone methylation data [12] available from the UCSC Genome Browser [55]. We used the UCSC table browser to intersect 15- or 11-bp alignments with predicted enhancers, and fed the results into GREAT [41] using the default settings, with an FDR-adjusted binomial *P*-value threshold of 0.05.

### Quantitative PCR

Twenty-five GFP expressing embryos were pooled for each injected construct (as well as an uninjected control pool) and dounce homogenized in TRIzol reagent (Life Technologies). Total RNA was extracted according to the manufacturer's instructions, and subsequently cleaned up using the RNeasy mini kit (Qiagen, Hilden, Germany). To reduce genomic DNA, we performed an on column digestion with the RNase-free DNase set (Qiagen). Total RNA (1.5 μg) was processed into cDNA using the High-Capacity cDNA Reverse Transcription Kit (Applied Biosystems, Foster City, CA, USA). For each reaction, we performed a control without the reverse transcriptase enzyme (no RT) to quantify residual genomic GFP. We performed Taqman assays for GFP or beta-actin 1 (*actb1*) using the TaqMan Universal PCR Master Mix, No AmpErase UNG reagent (Applied Biosystems), in triplicate. GFP expression was first normalized to the no RT conditions to single out GFP RNA, then to *actb1 *as a loading control.

## Abbreviations

bp: base pair; ChIP-seq: chromatin immunoprecipitation sequencing; DNase-seq: DNase I hypersensitive sites sequencing; FDR: false discovery rate; GFP: green fluorescent protein; H3K4me: histone 3, lysine 4 methylation; hpf: hours post-fertilization; MPRA: massively parallel reporter assay; MRCC: minimal reverse-complementary-covering; PCR: polymerase chain reaction; PWM: position weight matrix; RT: reverse transcriptase; TF: transcription factor; TFBS: transcription factor binding site; TSS: transcription start site.

## Authors' contributions

RPS, SJR, QL, KSP and NA conceived key aspects of the project and planned experiments. SJR, LO and KSP developed the underlying mathematical theory. SJR created and implemented the design algorithm. RPS, KKM, NMF and NO performed experiments. AKH, SJR, RPS and KSP analyzed the data and performed statistical analyses. RPS, SJR, KSP and NA wrote the manuscript. All authors commented on and revised the manuscript.

## Supplementary Material

Additional file 1 **Supplemental note**. Two results that justify and give a mathematical proof of correctness of our formal algorithm (in box), which we used to construct the oligomer library. Click here for file

Additional file 2 **Supplemental figures**. Seven supplemental figures and legends. Click here for file

Additional file 3 **Supplemental Table 1**. Multiplexed sequence screen data for 184 constructs tested using zebrafish transgenic assays. Click here for file

Additional file 4 **Supplemental Table 2**. Embryo counts from the functional dissection experiments. Click here for file

Additional file 5 **Supplemental Table 3**. Enriched gene ontology terms for 15-bp alignments of the four tissue-specific multiplexed oligomers. Click here for file

Additional file 6 **Supplemental Table 4**. Enriched gene ontology terms for 9-bp (+2 flanking bp) alignments of the three tissue-specific multiplexed oligomers. Click here for file

Additional file 7 **Supplemental Table 5**. Genomic position and embryo counts for the thirty approximately 1,000-bp regions tested for enhancer activity. Click here for file

## References

[B1] Euskirchen G Snyder M A plethora of sites. Nat Genet 2004 14 325 326 10.1038/ng0404-325 15054485

[B2] Badis G Berger MF Philippakis AA Talukder S Gehrke AR Jaeger SA Chan ET Metzler G Vedenko A Chen X Kuznetsov H Wang CF Coburn D Newburger DE Morris Q Hughes TR Bulyk ML Diversity and complexity in DNA recognition by transcription factors. Science 2009 14 1720 1723 10.1126/science.1162327 19443739 PMC2905877

[B3] Berger MF Philippakis AA Qureshi AM He FS Estep PW Bulyk ML Compact, universal DNA microarrays to comprehensively determine transcription-factor binding site specificities. Nat Biotechnol 2006 14 1429 1435 10.1038/nbt1246 16998473 PMC4419707

[B4] Visel A Rubin EM Pennacchio LA Genomic views of distant-acting enhancers. Nature 2009 14 199 205 10.1038/nature08451 19741700 PMC2923221

[B5] Loots GG Ovcharenko I rVISTA 2.0: evolutionary analysis of transcription factor binding sites. Nucleic Acids Res 2004 14 W217 221 10.1093/nar/gkh383 15215384 PMC441521

[B6] Lee D Karchin R Beer MA Discriminative prediction of mammalian enhancers from DNA sequence. Genome Res 2011 14 2167 2180 10.1101/gr.121905.111 21875935 PMC3227105

[B7] Narlikar L Sakabe NJ Blanski AA Arimura FE Westlund JM Nobrega MA Ovcharenko I Genome-wide discovery of human heart enhancers. Genome Res 2010 14 381 392 10.1101/gr.098657.109 20075146 PMC2840982

[B8] Heintzman ND Hon GC Hawkins RD Kheradpour P Stark A Harp LF Ye Z Lee LK Stuart RK Ching CW Ching KA Antosiewicz-Bourget JE Liu H Zhang X Green RD Lobanenkov VV Stewart R Thomson JA Crawford GE Kellis M Ren B Histone modifications at human enhancers reflect global cell-type-specific gene expression. Nature 2009 14 108 112 10.1038/nature07829 19295514 PMC2910248

[B9] Song L Zhang Z Grasfeder LL Boyle AP Giresi PG Lee BK Sheffield NC Graf S Huss M Keefe D Liu Z London D McDaniell RM Shibata Y Showers KA Simon JM Vales T Wang T Winter D Clarke ND Birney E Iyer VR Crawford GE Lieb JD Furey TS Open chromatin defined by DNaseI and FAIRE identifies regulatory elements that shape cell-type identity. Genome Res 2011 14 1757 1767 10.1101/gr.121541.111 21750106 PMC3202292

[B10] Giresi PG Kim J McDaniell RM Iyer VR Lieb JD FAIRE (Formaldehyde-Assisted Isolation of Regulatory Elements) isolates active regulatory elements from human chromatin. Genome Res 2007 14 877 885 10.1101/gr.5533506 17179217 PMC1891346

[B11] Sabo PJ Kuehn MS Thurman R Johnson BE Johnson EM Cao H Yu M Rosenzweig E Goldy J Haydock A Weaver M Shafer A Lee K Neri F Humbert R Singer MA Richmond TA Dorschner MO McArthur M Hawrylycz M Green RD Navas PA Noble WS Stamatoyannopoulos JA Genome-scale mapping of DNase I sensitivity in vivo using tiling DNA microarrays. Nat Methods 2006 14 511 518 10.1038/nmeth890 16791208

[B12] Aday AW Zhu LJ Lakshmanan A Wang J Lawson ND Identification of cis regulatory features in the embryonic zebrafish genome through large-scale profiling of H3K4me1 and H3K4me3 binding sites. Dev Biol 2011 14 450 462 10.1016/j.ydbio.2011.03.007 21435340 PMC3273848

[B13] Blow MJ McCulley DJ Li Z Zhang T Akiyama JA Holt A Plajzer-Frick I Shoukry M Wright C Chen F Afzal V Bristow J Ren B Black BL Rubin EM Visel A Pennacchio LA ChIP-Seq identification of weakly conserved heart enhancers. Nat Genet 2010 14 806 810 10.1038/ng.650 20729851 PMC3138496

[B14] Rada-Iglesias A Bajpai R Swigut T Brugmann SA Flynn RA Wysocka J A unique chromatin signature uncovers early developmental enhancers in humans. Nature 2011 14 279 283 10.1038/nature09692 21160473 PMC4445674

[B15] Visel A Blow MJ Li Z Zhang T Akiyama JA Holt A Plajzer-Frick I Shoukry M Wright C Chen F Afzal V Ren B Rubin EM Pennacchio LA ChIP-seq accurately predicts tissue-specific activity of enhancers. Nature 2009 14 854 858 10.1038/nature07730 19212405 PMC2745234

[B16] Ernst J Kheradpour P Mikkelsen TS Shoresh N Ward LD Epstein CB Zhang X Wang L Issner R Coyne M Ku M Durham T Kellis M Bernstein BE Mapping and analysis of chromatin state dynamics in nine human cell types. Nature 2011 14 43 49 10.1038/nature09906 21441907 PMC3088773

[B17] Myers RM Stamatoyannopoulos J Snyder M Dunham I Hardison RC Bernstein BE Gingeras TR Kent WJ Birney E Wold B Crawford GE A user's guide to the encyclopedia of DNA elements (ENCODE). PLoS Biol 2011 14 e1001046 10.1371/journal.pbio.1001046 21526222 PMC3079585

[B18] De Val S Chi NC Meadows SM Minovitsky S Anderson JP Harris IS Ehlers ML Agarwal P Visel A Xu SM Pennacchio LA Dubchak I Krieg PA Stainier DY Black BL Combinatorial regulation of endothelial gene expression by ets and forkhead transcription factors. Cell 2008 14 1053 1064 10.1016/j.cell.2008.10.049 19070576 PMC2782666

[B19] Visel A Minovitsky S Dubchak I Pennacchio LA VISTA Enhancer Browser--a database of tissue-specific human enhancers. Nucleic Acids Res 2007 14 D88 92 10.1093/nar/gkl822 17130149 PMC1716724

[B20] Schlabach MR Hu JK Li M Elledge SJ Synthetic design of strong promoters. Proc Natl Acad Sci USA 2010 14 2538 2543 10.1073/pnas.0914803107 20133776 PMC2823900

[B21] Ben-Dor A Karp R Schwikowski B Yakhini Z Universal DNA tag systems: a combinatorial design scheme. J Comput Biol 2000 14 503 519 10.1089/106652700750050916 11108476

[B22] Fordyce PM Gerber D Tran D Zheng J Li H DeRisi JL Quake SR De novo identification and biophysical characterization of transcription-factor binding sites with microfluidic affinity analysis. Nat Biotechnol 2010 14 970 975 10.1038/nbt.1675 20802496 PMC2937095

[B23] Compeau PE Pevzner PA Tesler G How to apply de Bruijn graphs to genome assembly. Nat Biotechnol 2011 14 987 991 10.1038/nbt.2023 22068540 PMC5531759

[B24] Mintseris J Eisen MB Design of a combinatorial DNA microarray for protein-DNA interaction studies. BMC Bioinformatics 2006 14 429 10.1186/1471-2105-7-429 17018151 PMC1635571

[B25] GitHub: sriesenfeld/MRCC-Libraries. https://github.com/sriesenfeld/MRCC-Libraries

[B26] Spector DJ Parks CL Knittle RA A multicomponent cis-activator of transcription of the E1b gene of adenovirus type 5. Virology 1993 14 128 136 10.1006/viro.1993.1242 8480416

[B27] Kawakami K Transgenesis and gene trap methods in zebrafish by using the Tol2 transposable element. Methods Cell Biol 2004 14 201 222 15602913 10.1016/s0091-679x(04)77011-9

[B28] Li Q Ritter D Yang N Dong Z Li H Chuang JH Guo S A systematic approach to identify functional motifs within vertebrate developmental enhancers. Dev Biol 2010 14 484 495 10.1016/j.ydbio.2009.10.019 19850031 PMC3330829

[B29] Ong CT Corces VG Enhancer function: new insights into the regulation of tissue-specific gene expression. Nat Rev Genet 2011 14 283 293 21358745 10.1038/nrg2957 PMC3175006

[B30] Gupta S Stamatoyannopoulos JA Bailey TL Noble WS Quantifying similarity between motifs. Genome Biol 2007 14 R24 10.1186/gb-2007-8-2-r24 17324271 PMC1852410

[B31] Bryne JC Valen E Tang MH Marstrand T Winther O da Piedade I Krogh A Lenhard B Sandelin A JASPAR, the open access database of transcription factor-binding profiles: new content and tools in the 2008 update. Nucleic Acids Res 2008 14 D102 106 10.1093/nar/gkn449 18006571 PMC2238834

[B32] Zhao Y Stormo GD Quantitative analysis demonstrates most transcription factors require only simple models of specificity. Nat Biotechnol 2011 14 480 483 21654662 10.1038/nbt.1893 PMC3111930

[B33] Chikh A Sayan E Thibaut S Lena AM DiGiorgi S Bernard BA Melino G Candi E Expression of GATA-3 in epidermis and hair follicle: relationship to p63. Biochem Biophys Res Commun 2007 14 1 6 10.1016/j.bbrc.2007.06.069 17632082

[B34] Kawahara A Nishi T Hisano Y Fukui H Yamaguchi A Mochizuki N The sphingolipid transporter spns2 functions in migration of zebrafish myocardial precursors. Science 2009 14 524 527 10.1126/science.1167449 19074308

[B35] Gotea V Visel A Westlund JM Nobrega MA Pennacchio LA Ovcharenko I Homotypic clusters of transcription factor binding sites are a key component of human promoters and enhancers. Genome Res 2010 14 565 577 10.1101/gr.104471.109 20363979 PMC2860159

[B36] Visel A Akiyama JA Shoukry M Afzal V Rubin EM Pennacchio LA Functional autonomy of distant-acting human enhancers. Genomics 2009 14 509 513 10.1016/j.ygeno.2009.02.002 19268701 PMC2683195

[B37] Swanson CI Evans NC Barolo S Structural rules and complex regulatory circuitry constrain expression of a Notch- and EGFR-regulated eye enhancer. Dev Cell 2010 14 359 370 10.1016/j.devcel.2009.12.026 20230745 PMC2847355

[B38] Thanos D Maniatis T Virus induction of human IFN beta gene expression requires the assembly of an enhanceosome. Cell 1995 14 1091 1100 10.1016/0092-8674(95)90136-1 8548797

[B39] Ho IC Leiden JM Regulation of the human T-cell receptor alpha gene enhancer: multiple ubiquitous and T-cell-specific nuclear proteins interact with four hypomethylated enhancer elements. Mol Cell Biol 1990 14 4720 4727 2388624 10.1128/mcb.10.9.4720-4727.1990 PMC361069

[B40] Small S Arnosti DN Levine M Spacing ensures autonomous expression of different stripe enhancers in the even-skipped promoter. Development 1993 14 762 772 8187640

[B41] McLean CY Bristor D Hiller M Clarke SL Schaar BT Lowe CB Wenger AM Bejerano G GREAT improves functional interpretation of cis-regulatory regions. Nat Biotechnol 2010 14 495 501 10.1038/nbt.1630 20436461 PMC4840234

[B42] Grskovic M Chaivorapol C Gaspar-Maia A Li H Ramalho-Santos M Systematic identification of cis-regulatory sequences active in mouse and human embryonic stem cells. PLoS Genet 2007 14 e145 10.1371/journal.pgen.0030145 17784790 PMC1959362

[B43] Sharon E Kalma Y Sharp A Raveh-Sadka T Levo M Zeevi D Keren L Yakhini Z Weinberger A Segal E Inferring gene regulatory logic from high-throughput measurements of thousands of systematically designed promoters. Nat Biotechnol 2012 14 521 530 10.1038/nbt.2205 22609971 PMC3374032

[B44] Nutiu R Friedman RC Luo S Khrebtukova I Silva D Li R Zhang L Schroth GP Burge CB Direct measurement of DNA affinity landscapes on a high-throughput sequencing instrument. Nat Biotechnol 2011 14 659 664 10.1038/nbt.1882 21706015 PMC3134637

[B45] Patwardhan RP Lee C Litvin O Young DL Pe'er D Shendure J High-resolution analysis of DNA regulatory elements by synthetic saturation mutagenesis. Nat Biotechnol 2009 14 1173 1175 10.1038/nbt.1589 19915551 PMC2849652

[B46] Melnikov A Murugan A Zhang X Tesileanu T Wang L Rogov P Feizi S Gnirke A Callan CG Jr Kinney JB Kellis M Lander ES Mikkelsen TS Systematic dissection and optimization of inducible enhancers in human cells using a massively parallel reporter assay. Nat Biotechnol 2012 14 271 277 10.1038/nbt.2137 22371084 PMC3297981

[B47] Patwardhan RP Hiatt JB Witten DM Kim MJ Smith RP May D Lee C Andrie JM Lee SI Cooper GM Ahituv N Pennacchio LA Shendure J Massively parallel functional dissection of mammalian enhancers in vivo. Nat Biotechnol 2012 14 265 270 10.1038/nbt.2136 22371081 PMC3402344

[B48] Hierholzer C Über die Möglichkeit, einen Linienzug ohne Wiederholung und ohne Unterbrechung zu umfahren. Mathematische Annalen 1873 14 30 32

[B49] Fisher S Grice EA Vinton RM Bessling SL Urasaki A Kawakami K McCallion AS Evaluating the biological relevance of putative enhancers using Tol2 transposon-mediated transgenesis in zebrafish. Nat Protoc 2006 14 1297 1305 10.1038/nprot.2006.230 17406414

[B50] Sprague J Bayraktaroglu L Bradford Y Conlin T Dunn N Fashena D Frazer K Haendel M Howe DG Knight J Mani P Moxon SA Pich C Ramachandran S Schaper K Segerdell E Shao X Singer A Song P Sprunger B Van Slyke CE Westerfield M The Zebrafish Information Network: the zebrafish model organism database provides expanded support for genotypes and phenotypes. Nucleic Acids Res 2008 14 D768 772 17991680 10.1093/nar/gkm956 PMC2238839

[B51] Zebrafish ENhancer browser. http://zen.ucsf.edu

[B52] The R Project for Statistical Computing. http://r-project.org

[B53] Sandelin A Wasserman WW Constrained binding site diversity within families of transcription factors enhances pattern discovery bioinformatics. J Mol Biol 2004 14 207 215 10.1016/j.jmb.2004.02.048 15066426

[B54] Langmead B Trapnell C Pop M Salzberg SL Ultrafast and memory-efficient alignment of short DNA sequences to the human genome. Genome Biol 2009 14 R25 10.1186/gb-2009-10-3-r25 19261174 PMC2690996

[B55] Dreszer TR Karolchik D Zweig AS Hinrichs AS Raney BJ Kuhn RM Meyer LR Wong M Sloan CA Rosenbloom KR Roe G Rhead B Pohl A Malladi VS Li CH Learned K Kirkup V Hsu F Harte RA Guruvadoo L Goldman M Giardine BM Fujita PA Diekhans M Cline MS Clawson H Barber GP Haussler D James Kent W The UCSC Genome Browser database: extensions and updates 2011. Nucleic Acids Res 2012 14 D918 923 10.1093/nar/gkr1055 22086951 PMC3245018

