# Supplementary material for: A compact, in vivo screen of all 6-mers reveals drivers of tissue-specific expression and guides synthetic regulatory element design
Source: Genome Biol. 2013 Jul 18;14(7):R72. doi: 10.1186/gb-2013-14-7-r72 (PMC4054837; doi:10.1186/gb-2013-14-7-r72)
Supplement: Additional file 1 — Supplemental note. Two results that justify and give a mathematical proof of correctness of our formal algorithm (in box), which we used to construct the oligomer library. [file gb-2013-14-7-r72-S1.DOCX]

***Smith, Riesenfeld et al. 2013***

***Additional File 1: Supplemental Note***

**Algorithm to Construct a Minimal Reverse-Complement-Covering (MRCC) Library**

Input: integers *k* and *m,* such that *k>*0 and ½*p_k_<m<*(½*n_k_*–¼*kp_k_*) (for *k=*2, 0*<m<*½*n_k_*).
*P*= the set of all *k*-mers that are palindromes

*G=*the (*k-*1)-dimensional de Bruijn graph

**Stage 1.** **Find disjoint paths between pairs of palindromes.**

1. Initialize the output sequence library to *L=*∅ and the current graph *H* to be *G* with an extra copy of the edges labeled by *P.*
2. Go to Stage 2 if *k*=2 or *k* is odd.
3. While there are still edges labeled by palindromes in *H*, repeat the following:
   1. Let *x*_1_ and *x*_2_ be distinct palindromes labeling edges in *H* such that *x*_2_ can be obtained by cyclically rotating the letters in *x*_1_.
   2. Compute a path *p*(*x*_1_, *x*_2_) from *x*_1_ to *x*_2_ in *H* that avoids other palindrome edges.
   3. Let *x*_1_*'* and *x*_2_*'* be the copies of *x*_1_ and *x*_2_*,* respectively.
      Compute a path from *x*_2_*'* to *x*_1_*'* which is reverse complementary to *p*(*x*_1_, *x*_2_).
   4. Remove both paths from *H.*
   5. Let *s* be the sequence that corresponds to the path *p*(*x*_1_, *x*_2_). Add *s* to *L.*

**Stage 2.** **Partition the remaining edges into reverse-complementary cycles.**

1. Initialize the cycle *C=*∅. While there are still edges in *H*, repeat the following:
2. Choose a vertex *u* such that: (1) if *C*≠∅ then *u* is in *C*, and (2) there exists an edge *e*=(*u*,*v*) in *H* directed out of vertex *u.*
3. Initialize the new path *D=*∅. Let *w*=*u* (*w* stores the first vertex of *D*).
4. While *D* is not a cycle, i.e., either *D* is empty or *u*≠*w*, repeat the following:
   1. Choose an edge *e* in *H* directed out of vertex *u* and into some vertex *v.*
   2. Remove *e* and its reverse complement from *H*.
   3. Add *e* to the current path *D*. Update the current vertex *u* by setting *u=v.*
5. If *C* is empty, set *C*=*D.* Otherwise, set the new value of *C* to be the cycle created by inserting cycle *D* into cycle *C* via vertex *u*.
6. Let *s* be the cyclical sequence corresponding to *C.* Divide *s* into *q*=*m*-½*p_k_* (*q=m* for *k=2*) consecutive sequences with *k*-1 bases of overlap at each cut.
7. Add these *q* sequences to *L*. Output *L*.

**Flanking-sequence-based, heuristic modification to Stage 2 (optional):**

1. Modify step A.iii.a: During periodic executions, select, if possible, an edge whose label begins (and/or ends) with the final 1–2 bases (initial 1–2 bases) of the left (right) flanking sequence. The period depends on the desired oligomer length.
2. Modify Step B:
   1. Optimize the cutting locations so that many resulting sequences of the desired length begin (and/or end) with the final (initial) 1–2 base pairs of the left (right) flanking sequence. Eliminate these “repeated” base pairs from such output sequences, and add the sequences to the library *L*.
   2. Patch together any remaining sequences (after the cutting) that are shorter than the desired oligomer length, and add the resulting oligomers to *L*.

Results 1 and 2 of this section justify and give a mathematical proof of correctness of our formal algorithm (in box), which we used to construct the oligomer library.

- ***Result 1.* For even values of *k, k>*2, no MRCC sequences exist, i.e., there is no analog of a de Bruijn sequence for *k*RC-pairs. Every MRCC library has size *m*>1.**

**Proof.** We denote by *f*(*x*) the reverse complement of a sequence *x*. We assume that there exists an MRCC sequence *s* of order *k*, and derive a contradiction. If *s* is an MRCC sequence, its reverse complement *f*(*s*) is also one. In the (*k-*1)-dimensional de Bruijn graph *G*, let *P* be the subset of edges that are labeled by palindromes.

Consider the multigraph *H* formed from adding the edges in the two paths corresponding to *s* and *f*(*s*) to the vertex set of *G* (Additional File 2)*.* The edges of *H* contain exactly one copy of each non-palindrome edge in *G* and two copies of each edge in *P.* The sequences *s* and *f*(*s*) correspond to a partitioning of the edges of *H* into two cycles such that one cycle is the reverse complement of the other. (One cycle contains one copy of the edges in *P* and the other cycle contains the second copy of the edges in *P*.)

We call an edge *e* that is labeled by a palindrome *x* *isolated* if there is no edge incident on an endpoint of *e* that is labeled by a distinct palindrome *x′*≠ *x.* If *k* is even and *k*>2, there are isolated edges in *G*. For a non-negative integer *i,* let *s^i^* denote the sequence formed from *i* consecutive repetitions of *s.* The edge labeled by the palindrome A^k/2^T^k/2^ (e.g., AAATTT for *k*=6) is isolated (Additional File 2).

Then let *e=*(*u,v*) be an isolated edge in *G.* In the multigraph *H*, there are two copies of *e* between vertices *u* and *v.* Since *e* is isolated, all other edges that are adjacent to *u* or *v* in *G* are not labeled by palindromes and hence have exactly one copy in *H*. In *G*, for every vertex *v*, the number of edges directed into *v* (*in*-*degree* of *v*) is equal to the number of edges directed out of *v* (*out*-*degree* of *v*). Thus, the extra copy of *e* in *H* causes the out-degree of *u* to be one greater than its in-degree, and the in-degree of *v* to be one greater than its out-degree. For example, for *k=*6, vertex AAATT has 4 incoming edges and 5 outgoing edges in *H* (Additional File 2).

For a directed (multi)graph to have a cycle decomposition, every vertex must have balanced degree. Since *H* has vertices with unbalanced degree, it does not have a cycle decomposition, which is a contradiction to the assumption that the edges of *H* can be partitioned into the cycles defined by *s* and *f*(*s*). ♦

- ***Result 2.* There is an efficient algorithm that constructs, for integers *k* and *m* such that *k>*0 and ½*p_k_<m<*(½*n_k_*–¼*kp_k_*) (for *k=*2, the range is 0*<m<*½*n_k_*), an MRCC library of order *k* and size *m.***

**Proof.** We show that each step in our algorithm (boxed text) is valid and can be executed in order, and that the output is an MRCC library of size *m.*

Let *k* and *m* be as prescribed. As in the proof of Result 1, let *G* be the (*k-*1)-dimensional de Bruijn graph and *P* be the subset of edges in *G* labeled by palindromes. (*P* is empty if *k* is odd.) Let *H* again be the multigraph created by adding to *G* a second copy of the edges in *P.* If an MRCC library *L* exists, then the set of reverse complementary sequences *f*(*L*) is also an MRCC library. *L* and its reverse complement *f*(*L*) correspond to a partitioning of the edges of *H* into two edge-disjoint sets of paths such that one set is the reverse complement of the other.

**Stage 1.** The proof of Result 1 above shows that the two copies of *P* in *H* cause an imbalance in the vertex degrees. Our algorithm resolves this issue: In Stage 1, for each pair of palindromes *x*_1_ and *x*_2_, the algorithm finds a path in *H* from *x*_1_ to *x*_2_ that does not go through any other palindrome edges. The reverse complement of this path is a path from *x*_2_ to *x*_1_, and the two paths in *H* correspond to a cycle in *G* (Additional File 2). Removing these paths from *H* eliminates both copies of the edges labeled by *x*_1_ and *x*_2_, and balances the degrees of the endpoints of those edges. We show that this can be done iteratively for every pair of palindromes, and that the edges that remain afterward can then be partitioned into two reverse-complementary cycles in Stage 2.

Our proof pairs the palindromes in a canonical way. It also gives a set of canonical, mutually edge-disjoint paths that connect each pair of palindromes. For a *k-*mer *x*=*b*_1_*b*_2_···*b_k_* and a positive integer *i*, let ρ(*x,i*) denote the *k*-mer obtained by cyclically rotating *x* to the left by i bases, i.e., ρ(*x,i*) = *b_i+_*_1_*b_i+_*_2_···b*_k_b*_1_*b*_2_···*b_i_*. For example, if *x=*AAATTT and *i*=2, then ρ(*x,i*)=ATTTAA. The *orbit* of *x*, denoted by ϕ(*x*), is the set of all *k*-mers that are cyclic rotations of *x*, that is, *y* is in ϕ(*x*) if there is some integer *i* such that *y*=ρ(*x,i*). For example, if *x=*AAATTT, then ϕ(*x*)={AAATTT, AATTTA, ATTTAA, TTTAAA, TTAAAT, TAAATT}.

Any palindrome *x* can be written as *x*=*zf*(*z*) (the concatenation of *z* and *f*(*z*)), where *z* is a sequence of length *k*/2. Simple algebra can show that the orbit ϕ(*x*_1_) of a palindrome *x*_1_ has the form {*x*_1_,*y*_1_,...,*y_r_*,*x*_2_*,f*(*y_r_*),...,f(*y*_1_)}, where *x*_2_ is a palindrome, the index *r* is at most *k*/2, and for all *i,* 1≤*i*≤*r*, *y_i_* is not a palindrome. Since there are exactly two palindromes in an orbit, ϕ defines a valid pairing of the palindromes: the pairs are those palindromes that share orbits. For example, for *k=*6, palindrome AAATTT is paired with palindrome TTTAAA.

For a pair of palindromes *x*_1_ and *x*_2_, let π(*x*_1_) denote the path labeled by the *k-*mers in ϕ(*x*_1_) that begins with the edge labeled by *x*_1_ and ends with the edge labeled by *x*_2_. Then these paths have the property that π(*x*_2_) is the reverse complement of π(*x*_1_), and the unique edges of π(*x*_1_)∪*f*(π(*x*_1_)) form a cycle in *G*. For example, if *x*_1_=AAATTT and *x*_2_=TTTAAA, then π(*x*_1_)={AAATTT, AATTTA, ATTTAA, TTTAAA}, and π(*x*_1_)={TTTAAA, TTAAAT, TAAATT, AAATTT} (Additional File 2).

Let *x*_1_ and *x*_3_ be palindromes such that *x*_3_ is not paired with *x*_1_, i.e., *x*_3_ is not in ϕ(*x*_1_). Then ϕ(*x*_3_)∩ϕ(*x*_1_)= ∅, which implies that π(*x*_1_) and π(*x*_3_) are edge disjoint. Since the paths defined by π from distinct orbits are pairwise edge-disjoint, the removal from *H* of the reverse-complementary paths defined by one orbit does not inhibit finding and removing the paths defined by another orbit. Hence, each iteration in Stage 1 can proceed, until *P* is empty.

**Note:** The canonical, orbit paths are useful for proving theoretically that Stage 1 can always be executed. However, the sequences that correspond to these paths vary in length (≥(*k*+1), ≤(*k*+*k*/2)). For most values of *m*, it is possible to equalize the lengths of all the sequences in the library *L,* by computing different palindrome paths. Care must be taken in computing non-canonical paths so that the paths for one pair of palindromes do not intersect with the paths for any other pair of palindromes, and the removal of these paths does not disconnect the graph. In practice, typical values of *m* do not cause difficulty because the graph is well connected. The constraints may become difficult to meet if *m* is very small (or, equivalently, if *L* is very large), relative to *N_k_*. Let *l*=*N_k_*/*m* be the desired length (so every sequence should have length *l* or *l*+1). For a pair of palindromes *x*_1_ and *x*_2_, one way to choose a non-canonical path π′(*x*_1_) from *x*_1_ to *x*_2_ of length *l-k*+1 is to take a random walk of *L-*2*k* edges from *x*_1_ to an arbitrary edge labeled by a *k-*mer *y* and then a direct path of *k*-1 edges from *y* to *x*_2_ (corresponding to the sequence formed by concatenating *y* and *x*_2_). The path π′(*x*_2_) from *x*_2_ to *x*_1_ is defined as *f*(π′(*x*_1_)). This is the version of the algorithm we implemented to produce the initial construct design.

**Stage 2.** To prove that Stage 2 can be successfully executed, we show first that just

after completion of Stage 1, the current graph *H* has the following two properties:

Stage 2 is an adaptation of the classic algorithm by Hierholzer for computing an Eulerian path. That algorithm initially computes a single cycle by choosing a starting vertex and following a trail of edges until it returns to the starting vertex (this is possible because the vertices have balanced degree). The cycle may not use every edge in the graph, however, in which case, because *G* is strongly connected (i.e., there is path in *G* between any pair of vertices), there is an unused edge out of some vertex *u* in the current cycle. A new cycle is found by following a new trail out of *u* until it returns to *u*. The new cycle is then spliced via vertex *u* into the original cycle. The process is repeated until all edges have been used. The major change in our version is that, for every edge followed, two edges are removed from the graph – the edge followed and its reverse complement ­– which raises a few new technical issues.

(1) Every vertex in *H* has balanced degree. Initially, every vertex in *G* has its in-degree equal to its out-degree. The set of edges removed from *H* in Stage 1 corresponds to a set of cycles in *G* (since *G* does not have the extra copy of palindrome edges). Removing a set of cycles does not change the degree balance of any vertex. But, *G* with those cycles removed is the same as *H* after Stage 1, so the claim of balanced degree also applies to *H*.

(2) *H* is strongly connected. Initially, *G* is strongly connected, implying that *H* also is. If *k* is odd, no edges are removed in Stage 1. If *k* is even, the edges removed from *H* in Stage 1 are all labeled by cyclic rotations of a palindrome. We show that for any two vertices *u* and *v*, there is a path from *u* to *v* in *G* that does not use any edge whose label is a cyclic rotation of a palindrome, implying that this path must still be present in *H* after Stage 1.

For any *k*-mer *y* that is in the orbit ϕ(*x*) of a palindrome *x*, it holds that, for every base *b*∈{A,T,G,C}, *b* and *f*(*b*) occur an equal number of times *y*. For any base *b*∈{A,T,G,C}, let *v_b_* denote the vertex whose label is *b^k^*^−1^, e.g., if *k=*6 and *b*=T, then *v_b_* is the vertex with label TTTTT. For a *k*-mer *x*=*a*_1_*a*_2_···*a_k_*, and a base *b*∈{A,T,G,C}, let σ(*x*,*b*) denote the *k-*mer obtained by shifting *x* once to the right and concatenating *b* on the end, i.e., σ(*x*,*b*) = *a*_2_*a*_3_···*a_k_b*. For example, if *x*=ATTTTT and *b*=G, then σ(*x*,*b*)=TTTTTG.

For any two bases *a,b*∈{A,T,G,C}, there is a path in *H* from *v_a_* to *v_b_*. If *b*≠*f*(*a*), then the path is labeled by the sequence of *k-*mers *x*_1_,...,*x_k_*_−1_, where *x*_1_=*a^k^*^−1^*b* and *x_i_*=σ(*x_i_*_−1_,*b*) for 2≤*i*≤*k*−1. Since each *k*-mer *x_i_* contains base *a* but not its complement *f*(*a*), it is not a cyclic rotation of a palindrome. Hence, the path is still in *H* after Stage 1. If *b*=*f*(*a*), choose a base *z*∈{A,T,G,C} such that *z* is not equal to *a* or *b.* Then the path in H from *v_a_* to *v_b_* is just the concatenation of the paths from *v_a_* to *v_z_* and from *v_z_* to *v_b_*.

Now let *u* and *w* be arbitrary vertices in *V*(G), and let *x_u_* and *x_w_* be their respective (*k*−1)-mer labels. Since *k* is even, *x_u_* and *x_w_* are sequences of odd length. Therefore, there must be bases *a,b*∈{A,T,G,C}, *a* occurs more than *f*(*a*) in *x_u_* and *b* occurs more than *f*(*b*) in *x_w_*. Then there is a path from *u* to *v_a_* labeled by the *k-*mers *x*_1_,...,*x_k_*_−1_ such that *x*_1_ = *x_u_a*, and *x_i_*=σ(*x_i_*_−1_,*a*) for 2≤*i*≤*k*−1. Since for each *x_i_*, *a* occurs more times than *f*(*a*) in *x_i_*, *x_i_* is not a cyclic rotation of a palindrome. Hence, the path is in *H*. By an analogous argument, there is a path in *H* from *v_b_* to w. The path in *H* from *u* to *w* is the concatenation of the paths from *u* to *v_a_*, *v_a_* to *v_b_*, and *v_b_* to *w*.

During the course of each iteration of Stage 2, step A, one cycle and its reverse complement (also a cycle) are removed from *H*. Since removing a cycle cannot unbalance the degrees of any vertices, each time when Step A is reached, the vertices in *H* have balanced degree.

**Step A.i.** Suppose at some iteration, step A.i cannot finish, i.e., there are still edges in *H*, but there is no edge out of a node already in the current cycle *C.* Since *H* is strongly connected prior to Stage 2, this implies that *C* is separated from the edges left in *H* by the reverse-complementary edges in *f*(*C*) that have been removed from *G* during Stage 2. In other words, every path that starts at a vertex in *C* and ends with an edge in *H* goes through at least one edge in *f*(*C*). Consider one such shortest such path *T*, which starts with a vertex *u* in *C* and consists of edges in *f*(*C*), except for the final edge *e*, which is in *H*. The first two edges in the reverse-complementary path *f*(*T*) are *f*(*e*)=(*v,w*), which must be in *H*, followed by the reverse complement of an edge in *C* that is directed out of vertex *w.* So *f*(*e*) is an edge in *H* that is directed into a vertex *w* that is in the cycle *C.* But we have argued that all vertices in the current graph *H* have balanced degree. Applying this condition to vertex *w* implies that there must also exist an edge *e*′=(*w,v*′) in *H* directed out of vertex *w*. Then *w* meets the requirements for a starting point, implying that Step A.i may be executed, which is a contradiction.

**Step A.iii.** We show that the inner loop in step A.iii always executes. The graph is unchanged between steps A.i and A.iii. Let *e*=(*u*,*v*) be the edge selected in step A.iii.a. Let *d* be the in-degree of *v* in *H* just prior to executing step A.iii.b. (Clearly, *d* is positive, since *e* is in *H*.) The loop in step A.iii can continue as long as the out-degree in *H* of vertex *v*, which is the last vertex in the current path, is positive.

In most circumstances, the out-degree of *v* is equal to *d* both before and after executing the next step A.iii.b. There are two cases, however, where the out-degree of *v* may be less than *d* after the execution. The first is if *v* is the start node *w*, in which case the cycle *D* is complete. So we now assume that *v*≠*w.* The second case is if the reverse-complementary edge *f*(*e*) that is removed from the graph in this step is itself directed out of vertex *v*, i.e., *f*(*e*) = (*v*,*y*) for some vertex *y*.

In this case, the (*k*-1)-mer labeling vertex *v* must be a palindrome (implying that *k* is odd or equal to 2). Consider whether either the current cycle *C* or the current path *D* already contains an edge *e*_1_ = (*u*_1_,*v*) into *v* and an edge *e*_2_ = (*v*,*u*_2_) out of *v* (if it contains one, it must contain the other). If there are no such edges in *C* or *D*, then the in- and out- degrees of *v* must still have their initial values of 4. Otherwise, the reverse-complementary cycle *f*(*C*) contains the edges *f*(*e*_1_) and *f*(*e*_2_). Since *v* is labeled by a palindrome, *f*(*e*_1_) is an edge out of *v*, and *f*(*e*_2_) is an edge into *v*. When edge *e*_1_ was removed from *H*, the simultaneous removal of the edge *f*(*e*_1_) from *H* balanced the degree of *v*: the in-degree and out-degree of *v* each decreased by 1. The analogous point is true regarding *e*_2_. Hence, just before step A.iii.b, the in- and out- degrees of vertex *v* are either both 4 or both 2. After the execution of this step, the in- and out- degrees of *v* are still positive and balanced (at either 3 or 1), and the loop of Step (c) can execute. (After the subsequent iteration of step A.iii the in- and out- degrees of *v* will once again be even and balanced, at either 2 or 0.) Thus, the the loop in Stage 2, step A.iii can continue until the path *C* is closed to form a cycle.

**Step A.iv.** The two cycles *C* and *D* have a common vertex *u*, so they can be composed in the standard way: rhe new cycle consists of the initial edges of *C*, up until vertex *u* is first reached; followed by the edges in cycle *D*, which start and finish at *u*; followed by the rest of cycle *C.*

**Step B.** Dividing the sequence corresponding to *C* into the appropriate number of sequences so that the total number in *L* is *m*. This is done in the standard way that maintains the coverage of *k*-mers*.* To check that the final set *L* is indeed an MRCC library, we note that, to create the paths which correspond to the sequences in *L*, each edge *e* in the initial multigraph *H* is traversed exactly once, at which point *e* and its reverse complement *f*(*e*) are both removed from the graph. This ensures that for each *k*RC-pair *X,* there is just one *k*-mer *x* in *X* that appears in *L*, and that *x* appears exactly once in *L*.

**Running Time and Space.** The algorithm can be implemented so that it runs in asymptotically optimal time and space *O*(*n_k_*), i.e. time and space that grow linearly with the size of the output set *L* (the *O* notation hides only small constants here). An explicit representation of *G* is not necessary for traversing the graph, since the edges are implicitly defined by the vertex labels. To prevent edges from being traversed more than once, a data structure such as the following can efficiently keep track of unused edges. Data for each of the *n_k_*_-1_ vertices are stored in an array so that a pointer to a vertex can be computed from its label sequence. For each vertex, its label is stored along with an array of 4 bits, corresponding to the 4 edges out of the vertex, which are ordered according to the last base in the edge label. A bit will be set to 0 if the corresponding edge is unused and 1 if it has been traversed. A global pointer to the current vertex is maintained, which allows the traversal to find the next unused edge in constant time. ♦
