# Supplementary material for: A compact, in vivo screen of all 6-mers reveals drivers of tissue-specific expression and guides synthetic regulatory element design
Source: Genome Biol. 2013 Jul 18;14(7):R72. doi: 10.1186/gb-2013-14-7-r72 (PMC4054837; doi:10.1186/gb-2013-14-7-r72)
Supplement: Additional file 2 — Supplemental figures. Seven supplemental figures and legends. [file gb-2013-14-7-r72-S2.DOCX]

***Smith, Riesenfeld et al. 2013***

***Additional File 2: Supplemental Figures and Legends***


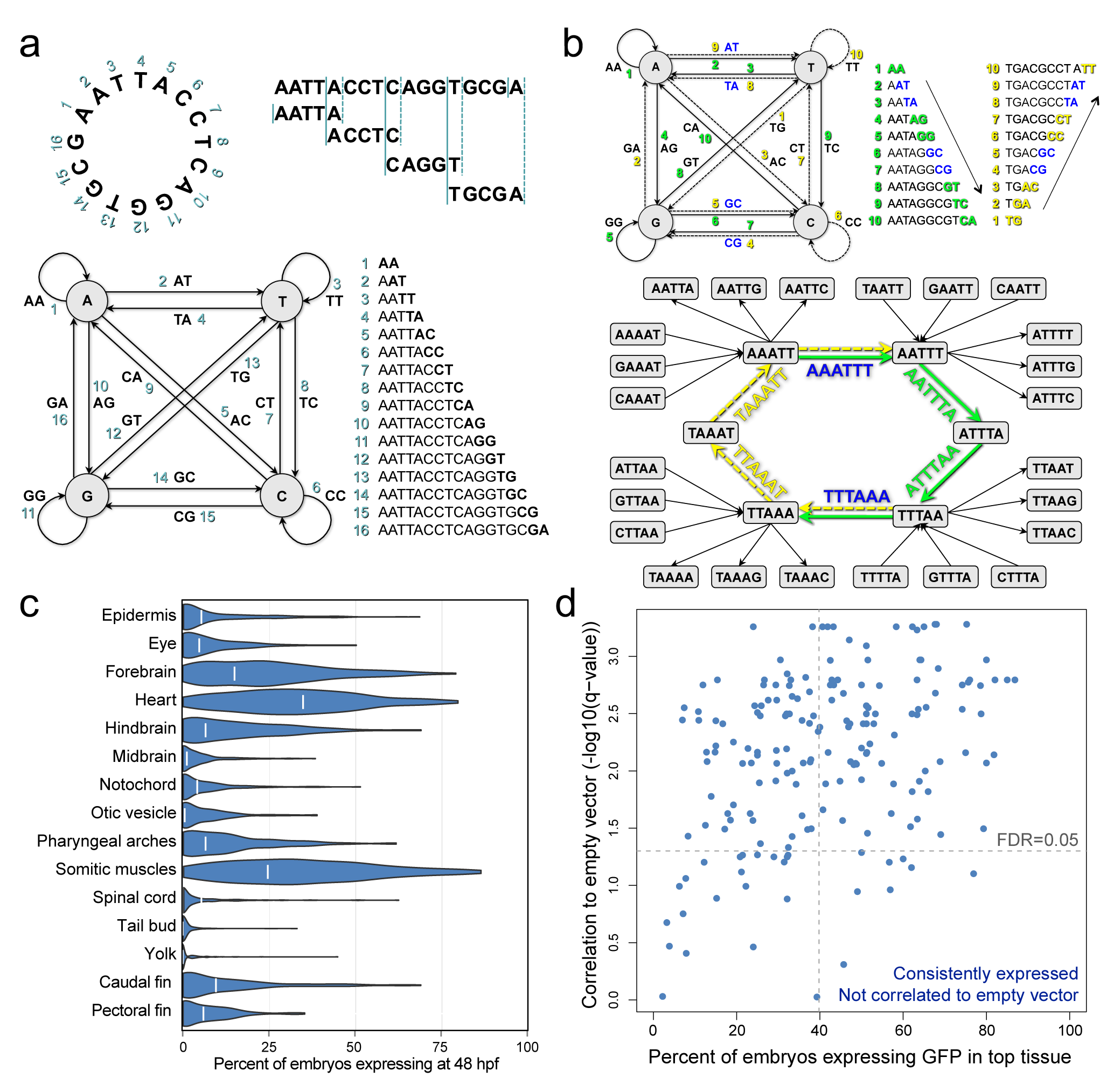


**Supplemental Figure 1**: (**a**) *Top:* A de Bruijn sequence of order *k* is a cyclical sequence in which every *k-*mer appears exactly once. A de Bruijn sequence of order 2 is shown on the left. On the right, the sequence is divided into four consecutive 5-bp oligomers such the last base of one sequence overlaps the first base of the next. The overlap preserves the coverage of all 2-mers among the oligomers. *Bottom:* The simple 1-dimensional de Bruijn graph has a vertex labeled by each monomer and an edge labeled by each 2-mer. The indices shown on the edges define an Eulerian cycle, i.e., a path that starts and ends at the same vertex and traverses every edge exactly once. The Eulerian cycle in the 1-dimensional de Bruijn graph corresponds to the de Bruijn sequence of order 2 given above. (**b**) *Top:* A multigraph *H* is created from a de Bruijn graph by adding a second copy of the edges labeled by self-reverse-complementary palindromes (Supplemental Experimental Procedures). For *k*=2, the palindrome edges (AT, TA, GC, and CG) form cycles in *H*. The edges of *H* can be partitioned into reverse-complementary cycles (green-indexed, solid edges and yellow-indexed, dotted edges), corresponding to MRCC sequences. *Bottom:* For *k*=6, a portion of *H* is shown, covering 30 of 1024 vertices and 30 of 4160 edges. Palindrome edges AAATTT and TTTAAA are isolated, i.e., neither endpoint is incident on a distinct palindrome edge. Their endpoints have unequal in- and out-degree (*e*.g., 4 edges are directed in and 5 edges are directed out of vertex AAATT). Therefore, for *k*=6, *H* cannot be decomposed into DNA-specific de Bruijn sequences. Our algorithm instead partitions the edges into reverse-complementary sets of paths to create an MRCC library. Cyclic rotations are used to define canonical pairs of palindromes, e.g., AAATTT and TTTAAA, and reverse-complementary paths between them (green solid edges and dashed yellow edges). Stage 1 of our algorithm iteratively removes pairs of canonical paths from *H*, while adding one oligomer for each pair to the library being constructed. (**c**) Violin plots depicting the distribution of the expression patterns of each tissue at 48 hpf. White lines indicate the fractional expression values for the empty vector construct. (**d**) Scatter plot depicting the method by which we selected consistently expressed multiplexed oligomers whose expression was not significantly correlated to minimal-promoter bias at 48hpf. The vertical dotted line denotes the 40% fractional expression threshold that was used, whereas the horizontal dotted line corresponds to an FDR-adjust p-value of 0.05.

**
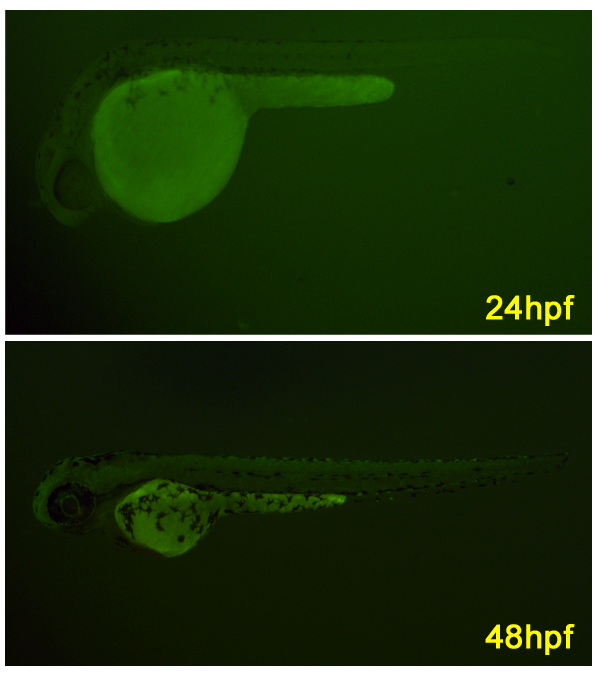
**

**Supplemental Figure 2:** Representative negative embryos injected with the E1b-tol2 empty vector and photographed at 24 and 48hpf.

**
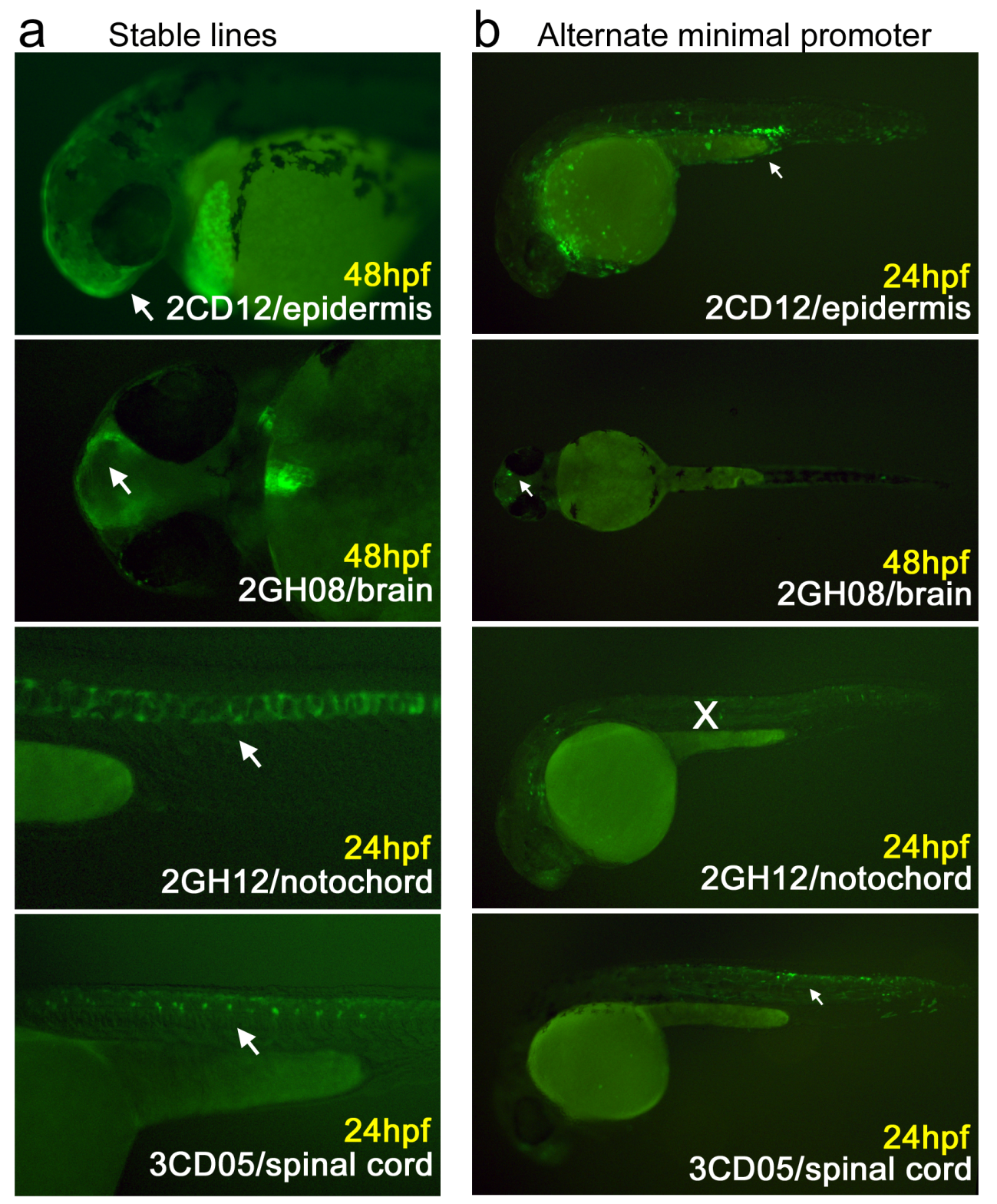
**

**Supplemental Figure 3**: **(a)** Stable F1 transgenics were generated by breeding wildtype Ab females with F0 males expressing 2CD12/epidermis, 2GH08/brain, 2GH12/notochord, and 3CD05/spinal cord. In each case, the F1 fish exhibited the original expression pattern (white arrows). **(b)** For each of the four tissue-specific multiplexed constructs, the E1b minimal promoter was replaced by a 31bp TATA-box containing minimal promoter from the pGL4.23 vector (Promega). In every case, except 2GH12/notochord, the pGL minimal promoter constructs exhibited the same tissue expression pattern (white arrows) as the original multiplexed sequence.

**
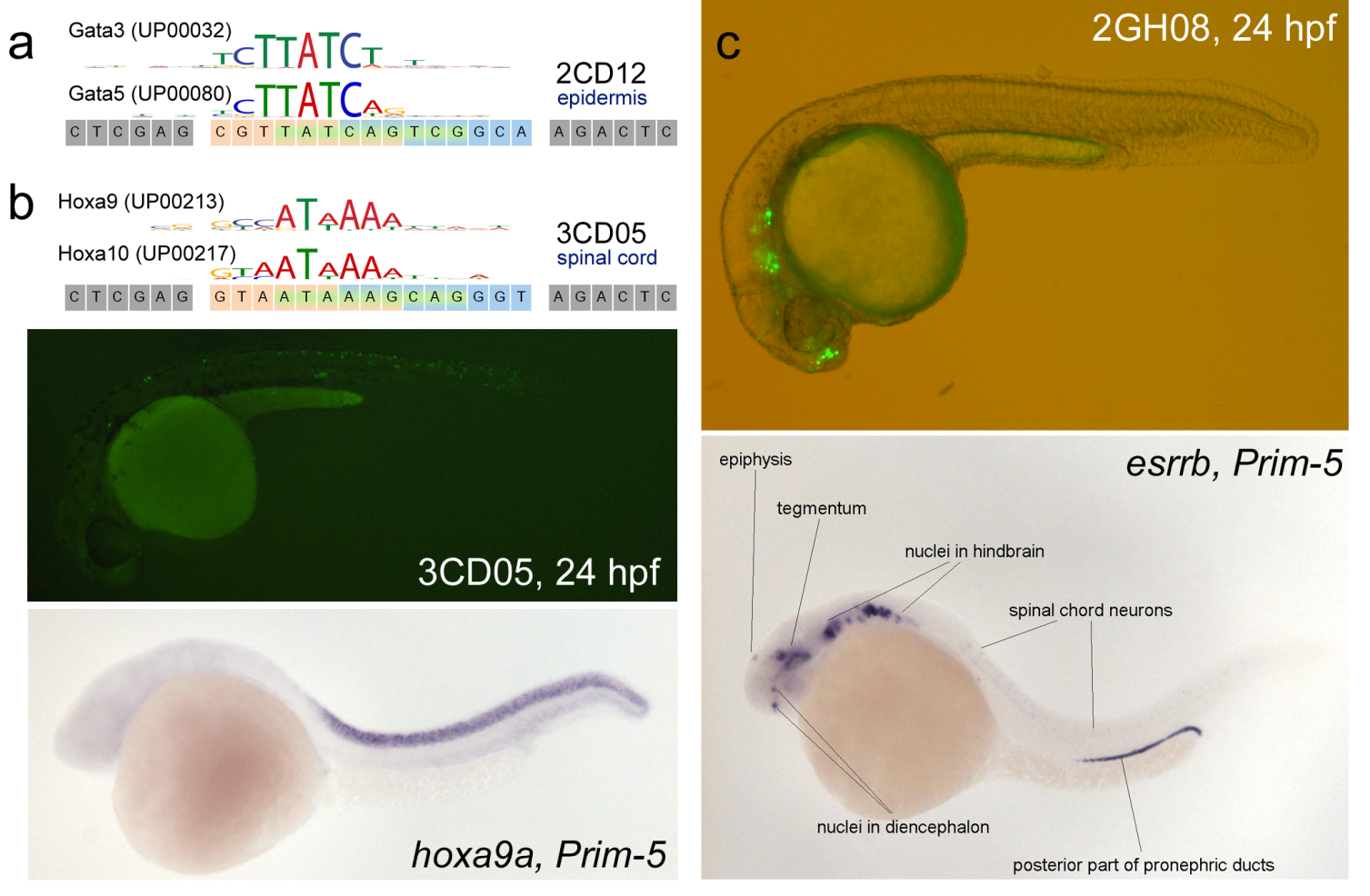
**

**Supplemental Figure 4**: **(a)** Comparison between the UniPROBE position weight matrix for Gata5 and Gata3. **(b)** *Top:* Comparison between the UniPROBE position weight matrix for Hoxa10 and Hoxa9. *Middle*: 3CD05 showing GFP expression in the spinal cord at 24hpf. *Bottom:* Whole-mount *in situ* hybridization for *hoxa9* at Prim-5 (~24 hpf) shows very specific staining in the spinal cord. Courtesy of zfin.org. **(c)** *Top*: Phase contrast/fluorescent overlay image of GFP driven by 2GH08 to the forebrain and lateral line ganglia. *Bottom*: Whole-mount *in situ* hybridization for *esrrb* at 24 hpf, which has a similar expression pattern. Labeling and figure provided courtesy of zfin.org.

**
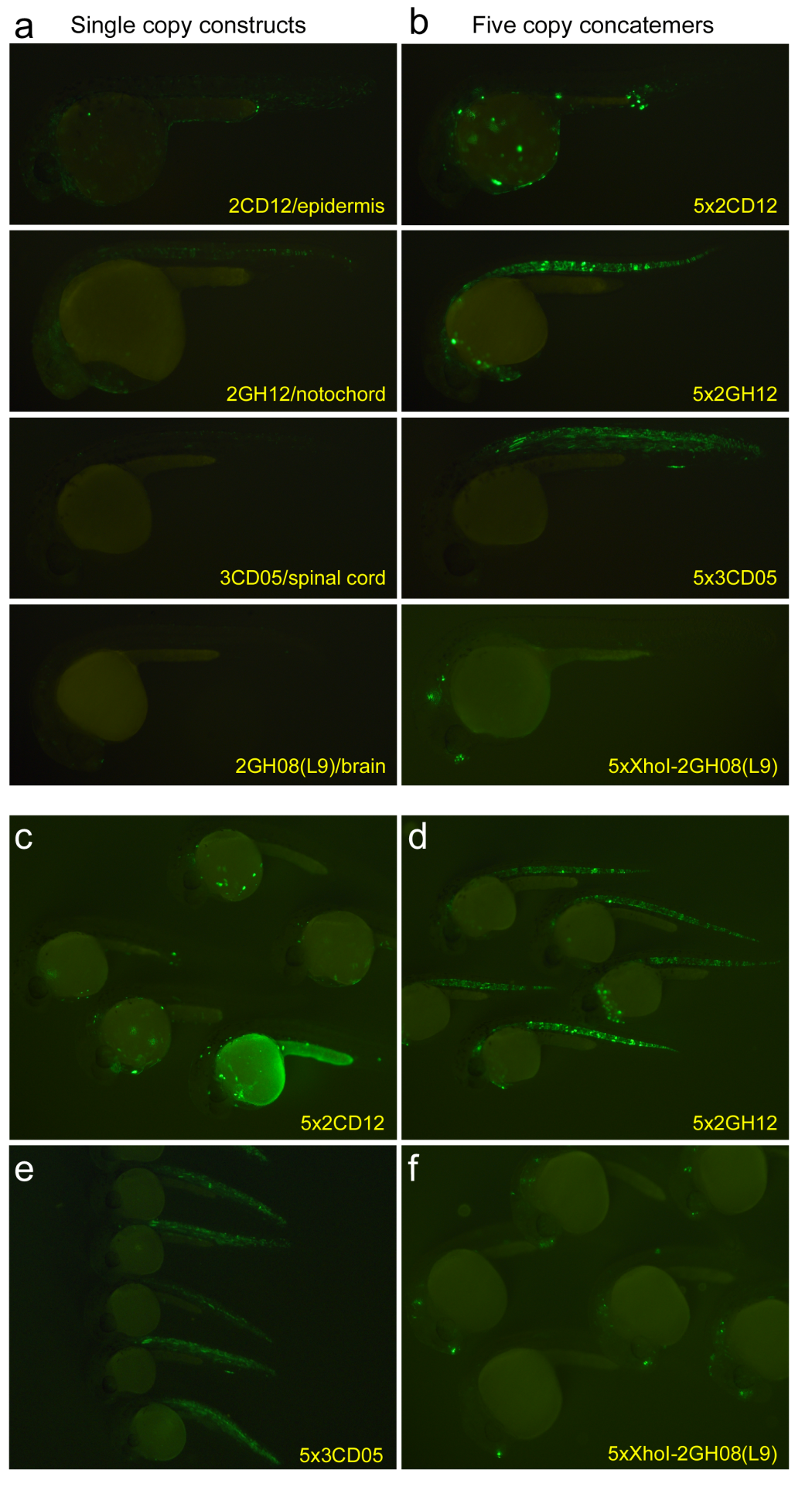
**

**Supplemental Figure 5**: Ungained images of embryos injected with **(a)** single copy multiplexed oligomers or **(b)** their 5x concatemer counterparts. Photos were taken using exactly the same camera and exposure settings. **(c-f)** Group images of embryos at 24 hpf exhibiting consistent, robust expression of the four concatemers in their respective tissues.


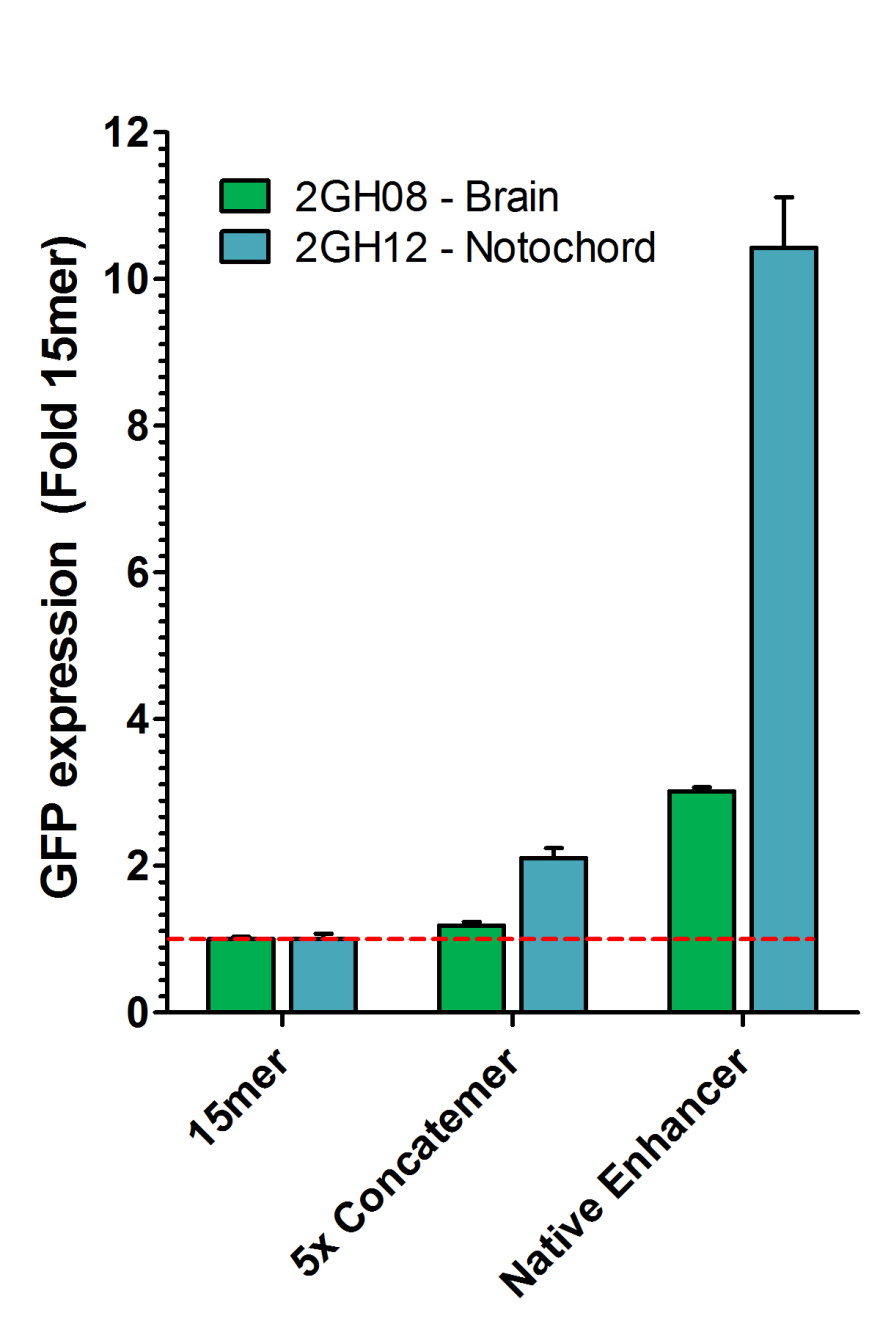


**Supplemental Figure 6**: Quantitative PCR (qPCR) analysis of synthetic constructs relative to native enhancers. Six pools of twenty five GFP-positive embryos expressing (i) 2GH08/brain or 2GH12/notochord, (ii) 5xXhoI-2GH08(L9) or 5x2GH12, or (iii) Fb2 or Nc2 were harvested at 24 hpf and assayed by Taqman qPCR assays for GFP and the housekeeping gene *actb1.* Expression is reported relative to the single 15-bp sequence, after normalizing to *actb1*. Error bars represent the standard deviation from technical replicates in a representative experiment. We did not observe any amplification of the GFP probe in an uninjected control pool of embryos.

**
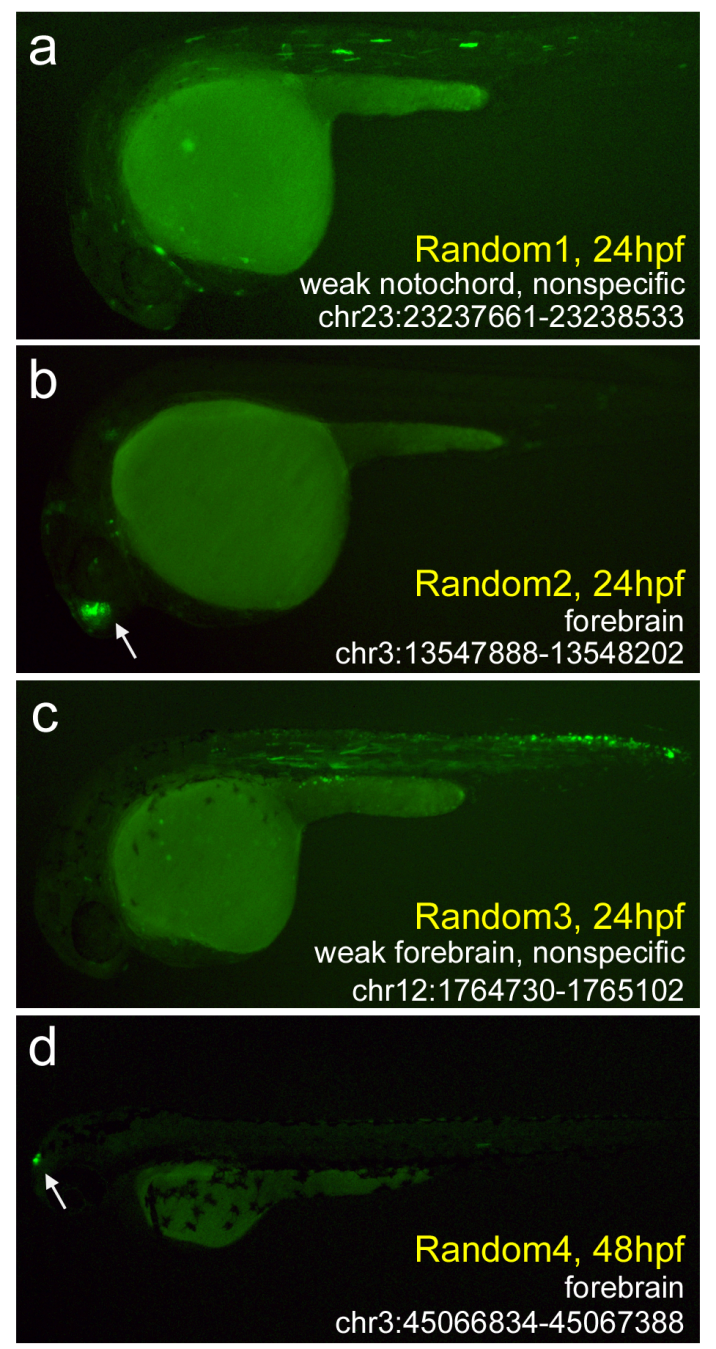
**

**Supplemental Figure 7**: Four positive enhancers that were chosen randomly (in Microsoft Excel) from a list of 35,045 H3K4me1+/me3- hotspots. **(a)** Random1 is the sole region to produce notochord expression (23/50 embryos at 24 hpf) and also produced considerable somitic muscle expression. **(b-d)** Three enhancers with forebrain expression. For two of these (Random 2 and 4), GFP expression was strong and specific. The third (Random 3) produced considerable expression in the somitic muscle and spinal cord, with marginal forebrain expression (18/45 embryos at 24 hpf). The complete data set is available in Additional File 7.
